# Unlocking the Diversity of Alkaloids in *Catharanthus roseus*: Nuclear Localization Suggests Metabolic Channeling in Secondary Metabolism

**DOI:** 10.1016/j.chembiol.2015.02.006

**Published:** 2015-03-19

**Authors:** Anna Stavrinides, Evangelos C. Tatsis, Emilien Foureau, Lorenzo Caputi, Franziska Kellner, Vincent Courdavault, Sarah E. O’Connor

**Affiliations:** 1 Department of Biological Chemistry, The John Innes Centre, Colney, Norwich NR4 7UH, UK; 2 Université François Rabelais de Tours, EA2106 “Biomolécules et Biotechnologies Végétales”, 37200 Tours, France

## Abstract

The extraordinary chemical diversity of the plant-derived monoterpene indole alkaloids, which include vinblastine, quinine, and strychnine, originates from a single biosynthetic intermediate, strictosidine aglycone. Here we report for the first time the cloning of a biosynthetic gene and characterization of the corresponding enzyme that acts at this crucial branchpoint. This enzyme, an alcohol dehydrogenase homolog, converts strictosidine aglycone to the heteroyohimbine-type alkaloid tetrahydroalstonine. We also demonstrate how this enzyme, which uses a highly reactive substrate, may interact with the upstream enzyme of the pathway.

## Highlights

• Tetrahydroalstonine synthase catalyzes the formation of a plant-derived alkaloid
• Tetrahydroalstonine synthase is localized to the nucleus
• Tetrahydroalstonine synthase and the preceding pathway enzyme interact
• Discovery of a gene controlling structural diversity of monoterpene indole alkaloids

## Graphical Abstract

**Figure:**
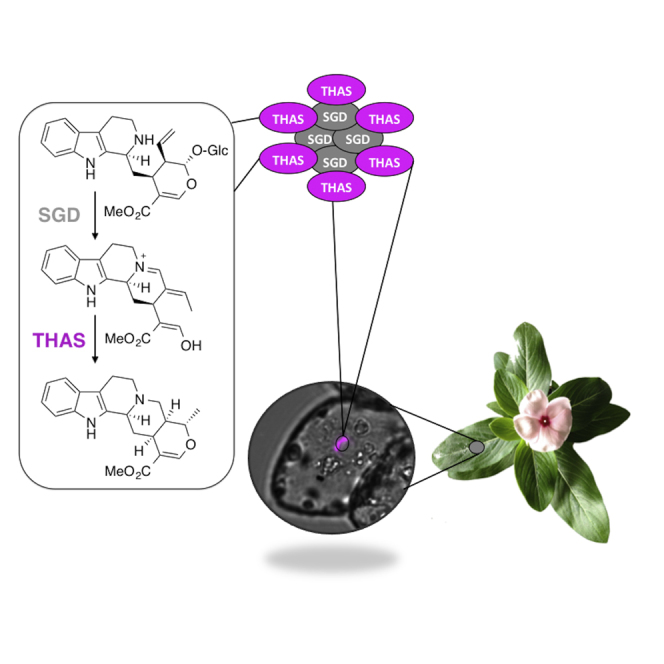

## Teaser

How plants transform the central biosynthetic intermediate strictosidine into thousands of divergent alkaloids has remained unresolved. Stavrinides et al. discover a nuclear-localized alcohol dehydrogenase homolog responsible for conversion of strictosidine aglycone to tetrahydroalstonine that appears to interact with an upstream pathway enzyme.

## Introduction

The monoterpene indole alkaloids (MIAs) are a highly diverse family of natural products that are produced in a wide variety of medicinal plants. Over 3000 members of this natural product class, which includes compounds such as quinine, vinblastine, reserpine, and yohimbine, are derived from a common biosynthetic intermediate, strictosidine aglycone (O'Connor and Maresh, 2006). How plants transform strictosidine aglycone into divergent structural classes has remained unresolved.

**Figure 1:**
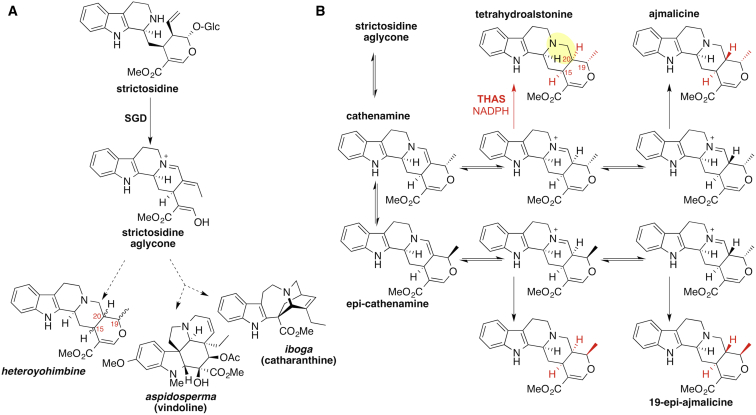
The Monoterpene Indole Alkaloids (A) Representative monoterpene indole alkaloids derived from strictosidine and strictosidine aglycone found in *Catharanthus roseus*. (B) Heteroyohimbine biosynthesis.

The recent availability of transcriptome and genome data has dramatically accelerated the rate at which new plant biosynthetic genes are discovered. All genes that lead to strictosidine aglycone have been recently cloned from the well-characterized medicinal plant *Catharanthus roseus*, which produces over 100 MIAs (De Luca et al., 2014). However, gene products that act on strictosidine aglycone have not been identified in any plant, despite decades of effort. Attempts have been hampered in part by the reactivity and instability of strictosidine aglycone. In *C. roseus*, there are at least two major pathway branches derived from strictosidine aglycone (O'Connor and Maresh, 2006). One pathway is hypothesized to lead to the aspidosperma and the iboga classes to yield the precursors of vinblastine, while the other is expected to lead to alkaloids of the heteroyohimbine type (Figure 1A). These alkaloids have diverse biological activities: vinblastine is used as an anticancer agent (Kaur et al., 2014) and the heteroyohimbines have a range of pharmacological uses (Costa-Campos et al., 1998; Elisabetsky and Costa-Campos, 2006). While it is unknown how many *C. roseus* enzymes use strictosidine aglycone as a substrate, there is clearly more than one enzyme that acts at this crucial branchpoint.

The biochemical pathway leading from strictosidine aglycone to the heteroyohimbine alkaloids has been previously investigated using both crude plant extracts and biomimetic chemistry. Reduction of strictosidine aglycone with NaBH_4_ or NaCNBH_3_ yielded the heteroyohimbines ajmalicine (raubasine), tetrahydroalstonine, and 19-epi-ajmalicine, which differ only in the stereochemical configuration at carbons 15, 19, and 20, in various ratios (Figure 1B) (Brown et al., 1977; Kan-Fan and Husson, 1978, 1979, 1980). These three diastereomers were again observed, also in varying relative amounts, when crude *C. roseus* protein extracts were incubated with strictosidine aglycone and NADPH, but not in the absence of NADPH (Rueffer et al., 1979; Stoeckigt et al., 1976, 1977, 1983; Zenk, 1980). Collectively, these observations indicate that the heteroyohimbines result directly from the reduction of strictosidine aglycone and that an NADPH-dependent enzyme is implicated in this process. However, no gene encoding such an enzyme has been identified. Here we report the discovery of a reductase that converts strictosidine aglycone to the heteroyohimbine alkaloid tetrahydroalstonine.

## Results and Discussion

**Figure 2:**
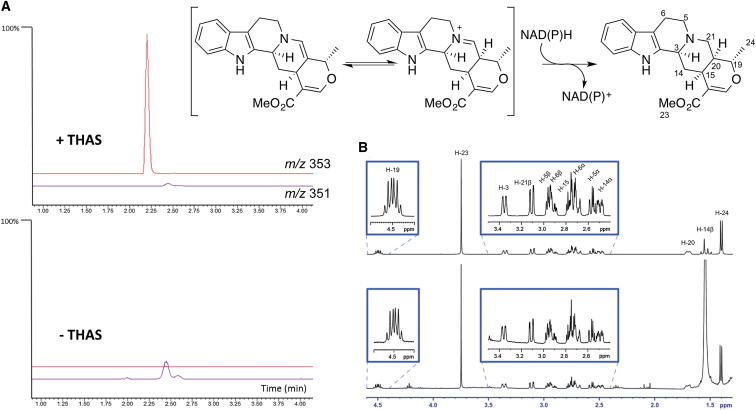
Activity Assays of THAS Enzyme reactions were performed at 25°C for 30 min and assayed using a mass spectrometer in tandem with ultraperformance liquid chromatography. (A) The total ion chromatogram for *m*/*z* 353 (red trace) and *m*/*z* 351 (purple trace) from 1 to 4 min is shown. Top trace: THAS (50 nM), SGD (6 nM), strictosidine (200 μM), NADPH (200 μM); bottom trace: same reaction in the absence of THAS. The *y* axis represents normalized ion abundance as a percentage relative to 1.00e^8^ detected by selected ion monitoring at *m*/*z* 353 and 351. (B) Portion of the ^1^H-NMR spectrum of the isolated enzymatic product compared with an authentic standard of tetrahydroalstonine.

Given that heteroyohimbine biosynthesis likely requires reduction of an iminium present in strictosidine aglycone (Figure 1B), we used a publically available RNA-seq database that we recently generated (Gongora-Castillo et al., 2012) to search for *C. roseus* candidates displaying homology to enzyme classes known to reduce the carbonyl functional group. The alcohol dehydrogenases (ADHs), enzymes that reduce aldehydes and ketones to alcohols, were chosen as the initial focus. As part of a screen of ADHs that are upregulated in response to methyl jasmonate (Gongora-Castillo et al., 2012), a hormone known to upregulate alkaloid biosynthesis, we identified a candidate annotated as sinapyl alcohol dehydrogenase (Supplemental Information). When heterologously expressed and purified from *E. coli* (Figure S1), and assayed with strictosidine aglycone and NADPH, this candidate yielded a product with a mass consistent with a heteroyohimbine (*m*/*z* 353.1855), thereby implicating this enzyme in the important structural branchpoint of the MIA biosynthetic pathway (Figure 2A).

**Figure 3:**
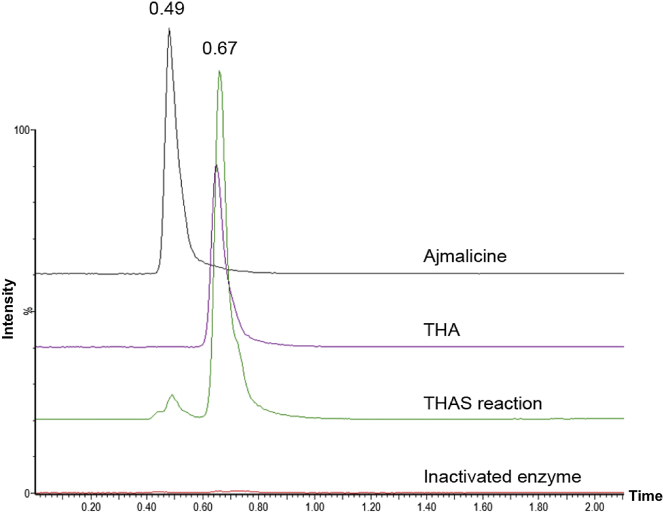
LC-MS Performed under Normal Phase Conditions (Hydrophilic Interaction Liquid Chromatography) Showing Separation of Ajamlicine (Retention Time of 0.49 min) and Tetrahydroalstonine (THA, Retention Time 0.67 min) THAS produces approximately 95% of the tetrahydroalstonine (THA) diastereomer. The *y* axis represents normalized ion abundance as a percentage detected by selected ion monitoring at *m*/*z* 353.

To determine the identity of the alkaloid product, the enzyme was incubated with purified strictosidine (4.3 mg) in the presence of strictosidine glucosidase (SGD), which generated strictosidine aglycone in situ to best mimic physiologically relevant conditions. The major product (approximately 1 mg) was isolated by preparative thin-layer chromatography and exhibited an ^1^H-NMR and ^13^C-NMR spectrum matching an authentic standard of tetrahydroalstonine (Figure 2B; Figure S2). Hemscheidt and Zenk (1985) previously reported the isolation of an enzyme that produced tetrahydroalstonine, although this protein was purified only 35-fold from *C. roseus* cell cultures. Consistent with Hemscheidt and Zenk’s (1985) nomenclature, we named this enzyme tetrahydroalstonine synthase (THAS). A minor enzymatic product was produced in yields too low for NMR characterization, but had a mass and *R*_f_ value consistent with ajmalicine, a stereoisomer of tetrahydroalstonine (Figure S2). When applied to normal phase liquid chromatography conditions, ajmalicine and tetrahydroalstonine could be resolved, indicating that the enzyme produces approximately 95% tetrahydroalstonine (Figure 3; Supplemental Information). We also silenced this gene in *C. roseus* seedlings using virus-induced gene silencing (VIGS) (Liscombe and O'Connor, 2011). LC-mass spectrometry (MS) analysis of the silenced leaf tissue showed a statistically significant decrease (approximately 50%) of a peak with a mass and retention time consistent with a heteroyohimbine, suggesting that this enzyme is involved in this biosynthetic pathway branch in vivo (Figure S2). A 50% reduction in product levels upon silencing has been observed for other physiologically relevant biosynthetic genes using the VIGS approach in both *C. roseus* (Asada et al., 2013; Geu-Flores et al., 2012) and another well-studied medicinal plant, opium poppy (Desgagne-Penix and Facchini, 2012; Chen and Facchini, 2014). Therefore, THAS is likely a major producer of tetrahydroalstonine in vivo, although additional, undiscovered *C. roseus* enzymes could also contribute to production of this compound. While we could not resolve tetrahydroalstonine and its stereoisomer ajmalicine in the silenced crude extracts, the levels of the ajmalicine-derived alkaloid serpentine remain the same, suggesting that silencing of THAS does not substantially affect ajmalicine levels and consequently that THAS does not play a major role in the biosynthesis of ajmalicine in planta.

Small-scale assays using LC-MS to monitor product formation indicated that NADPH was required for the reaction, although NADH could also be utilized (Figure S1). Efforts to accurately measure the steady state kinetic constants of this enzyme were complicated because strictosidine aglycone reacts with nucleophiles, opening the possibility that the substrate reacts with components in the reaction or the enzyme. This reactivity has already been associated with a plant defense mechanism involving strictosidine aglycone-mediated aggregation of proteins in *C. roseus* (Guirimand et al., 2010). Nevertheless, we obtained estimated *K*_m_ and *k*_cat_ values (Figure S1). To support these kinetic data, we also performed isothermal titration calorimetry (ITC) with THAS in the presence of NADPH and strictosidine aglycone. Titration of THAS with NADPH indicated that the cosubstrate binds first with a *K*_d_ of 1.5 ± 0.1 μM (Δ*H* (cal/mol) 2310 ± 123.2; Δ*S* (cal/mol/deg) 34.2 ± 0.3) (Figure S1). The aglycone substrate does not appear to bind in the absence of NADPH, suggesting that the enzyme utilizes an ordered binding mechanism in which NADPH binds first. However, titration of the THAS-NADPH complex with strictosidine aglycone led to formation of a precipitate when concentrations of strictosidine aglycone exceeded 60 μM, preventing calculation of an accurate *K*_d_. Collectively, the ITC data for THAS are consistent with an ordered Bi-Bi mechanism, a kinetic mechanism that has been reported for similar ADHs such as cinnamyl alcohol dehydrogenase (Charlier and Plapp, 2000; Lee et al., 2013).

The amino acid sequence of THAS was subjected to a BLAST alignment against the *C. roseus* transcriptome (Gongora-Castillo et al., 2012), as well as the NCBI (Figure S3). The closest characterized homologs of THAS are sinapyl alcohol dehydrogenase (*Populus tremuloides*, 64% amino acid identity), cinnamyl alcohol dehydrogenase (*Populus tomentosa*, 64%) and 8-hydroxygeraniol dehydrogenase (*C. roseus*, 63%), which are zinc-containing medium chain ADHs (Bomati and Noel, 2005; Lee et al., 2013).

Strictosidine aglycone can rearrange into several isomers (Figure 1B), and while it has been reported that the dominant isomer is cathenamine (Gerasimenko et al., 2002; Stoeckigt et al., 1977), equilibration in solution with other isomers occurs (Brown and Leonard, 1979; Stoeckigt et al., 1983). Reduction of cathenamine or epi-cathenamine (Figure 1B) by a reductase would require reduction of the carbon-carbon double bond of an enamine; alternatively, Stoeckigt et al. (1983) and Zenk (1980) suggested that the iminium isomer is reduced (Figure 1B). THAS may catalyze the stereoselective formation of tetrahydroalstonine by selectively binding the correct isomer of the substrate for reduction, thereby relying on the inherent propensity for the enamine and imine to tautomerize under physiological conditions. Given that three diastereomers, ajmalicine, tetrahydroalstonine, and 19-epi-ajmalicine, can be obtained from chemical reduction of strictosidine aglycone, this is a chemically reasonable proposal. An alternative hypothesis is that THAS catalyzes enamine-imine tautomerization in addition to reduction. The difficulties associated with obtaining accurate kinetic data in this system, as well as the inherent reactivity of the strictosidine aglycone, make answering these questions using enzymology approaches challenging. However, identification and comparison with enzymes that generate other heteroyohimbine diastereomers will likely provide the basis for a more definitive mechanism of product specificity.

**Figure 4:**
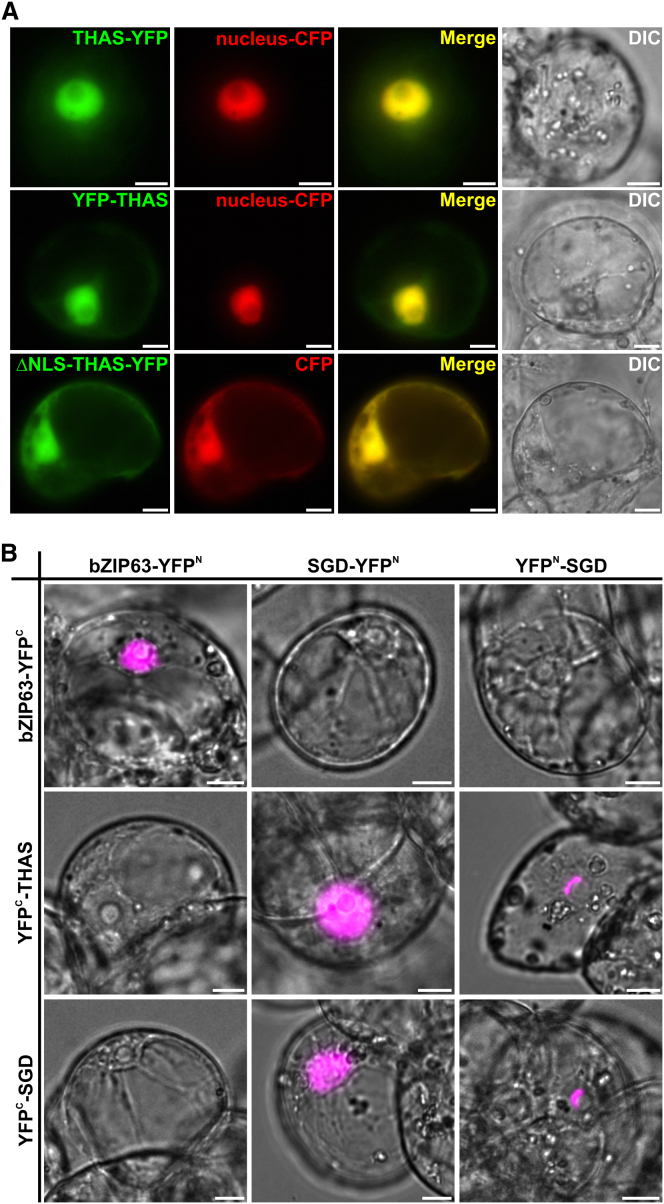
THAS Is Targeted to the Nucleus via a Monopartite Nuclear Localization Signal (NLS) and Interacts with SGD (A) *C. roseus* cells were transiently cotransformed with plasmids expressing either THAS-YFP (upper row), YFP-THAS (middle row), or the NLS deleted version of THAS (lower row) and plasmids encoding the nuclear CFP marker or the nucleocytosolic CFP marker (second column). Colocalization of the fluorescence signals appears in yellow when merging the two individual (green/red) false color images (third column). Cell morphology is observed with differential interference contrast (DIC) (fourth column). (B) THAS and SGD interactions were analyzed by BiFC in *C. roseus* cells transiently transformed by plasmids encoding fusions indicated on the top (fusion with the split YFP^N^ fragment) and on the left (fusion with split YFP^C^ fragment). bZIP63 was used as a positive BiFC control and to evaluate the specificity of THAS and SGD interactions. The images are merges of the YFP BiFC channel (magenta false color) with the DIC channel to show the nuclear localization of the interactions. Bars, 10 μm.

Recent research has highlighted that plant secondary metabolite biosynthetic pathways often are compartmentalized in different subcellular locations. While microscopy experiments have demonstrated that most of the early steps of monoterpene indole alkaloid biosynthesis in *C. roseus* take place in the cytosol (Courdavault et al., 2014), the enzyme that synthesizes strictosidine is located in the vacuole, and the enzyme SGD, which deglycosylates strictosidine, contains a nuclear localization signal and is in the nucleus, a highly unusual site for secondary metabolite biosynthesis (Guirimand et al., 2010). Notably, a motif resembling a class V nuclear localization sequence (Kosugi et al., 2008) was observed in THAS (K_214_K_215_K_216_R_217_). Microscopy of *C. roseus* cells transformed with YFP-tagged THAS confirmed the nuclear location of this enzyme, while deletion of the KKKR sequence disrupted the localization (Figure 4A; Figure S4). This is one of the very few examples of secondary metabolism that is localized to the nucleus (Saslowsky et al., 2005).

Given the reactivity of strictosidine aglycone (Guirimand et al., 2010), metabolic channeling via a protein-protein interaction between SGD and the enzyme immediately downstream may be necessary to protect the substrate. Pull down experiments between SGD and THAS gave partially positive but inconclusive results (Figure S4). However, when we used bimolecular fluorescence complementation (BiFC) in *C. roseus* cells, we observed an interaction between SGD and THAS (Figure 4B). While this interaction generated a diffuse nuclear fluorescent signal when the C-terminal end of SGD was fused to the split-YFP fragment, a sickle-shaped signal was observed when both SGD and THAS were expressed with free C-terminal ends (YFP^N^-SGD and YFP^C^-THAS). Such a signal was also observed for SGD self-interactions (Guirimand et al., 2010) and likely results from the formation of SGD complexes over 1.5 MDa (Luijendijk et al., 1998). Similar experiments with SGD and an upstream MIA biosynthetic enzyme, loganic acid methyl transferase, failed to show an interaction, highlighting the specificity of this interaction (Figure S4). The fact that THAS interacts with SGD provides further support for the physiological relevance of THAS in planta. As strictosidine aglycone is reactive and most likely toxic in vivo, it has been proposed that this molecule is produced by the plant in response to attack (Guirimand et al., 2010). The nuclear localization of THAS might be an evolutionary mechanism designed to channel this molecule into a more stable product when no such defense is required. Identification of additional nuclear-localized biosynthetic enzymes in *C. roseus* and other heteroyohimbine producing plants may provide more insight into the reasons for this unusual localization pattern.

## Significance

**Many of the monoterpene indole alkaloid structural classes are generated at the SGD junction. Here we report the first identification of a biosynthetic gene that acts directly downstream of SGD. The enzyme, an ADH homolog, generates a heteroyohimbine alkaloid by reducing one of the isomers of strictosidine aglycone. Unusually, this enzyme is located in the nucleus and may interact with its upstream partner, SGD. The discovery of the THAS gene represents the completion of a major branch of monoterpene indole alkaloid biosynthesis, which will now allow reconstruction of heteroyohimbines and heteroyohimbine analogs in heterologous hosts. This discovery is a crucial first step in understanding how the structural diversity of MIAs is controlled.**

## Experimental Procedures

The THAS gene (accession number KM524258) was cloned into pOPINF and expressed in Rosetta 2 pLysS *E. coli* cells (Novagen) with induction of expression with 0.1 mM isopropyl β-D-1-thiogalactopyranoside. Cultures were grown at 18°C for 16 hr, with shaking at 200 rpm. His-tagged THAS was purified using a HisTrap FF 5-ml column (GE Healthcare). SGD expression and purification was done as described for THAS using the expression system described previously by Yerkes et al. (2008). Purified THAS and SGD were used in all assays. Strictosidine was enzymatically synthesized from tryptamine and a crude methanol extract of snowberries (*Symphoricarpos albus*) enriched in secologanin prepared as previously described (Geerlings et al., 2001). Strictosidine aglycone was generated in situ prior to addition of THAS by incubation of strictosidine and SGD in the appropriate solution for 10 min, at which time strictosidine was completely converted to the aglycone.

Steady state kinetic analyses were performed with 50 nM THAS and 6 nM SGD, 50 mM phosphate buffer (pH 7.5), 200 μM NADPH, and an internal caffeine standard (50 μM). All LC-MS measurements were performed on AQUITY ultraperformance liquid chromatography with a Xevo TQ-S mass spectrometer.

For VIGS, a 330-bp fragment of THAS was cloned into the pTRV2u vector as described (Geu-Flores et al., 2012). The resulting pTRV2u-THAS construct was used to silence THAS in *C. roseus* seedlings essentially as described (Liscombe and O'Connor, 2011).

The subcellular localization of THAS was studied by creating fluorescent fusion proteins using the pSCA-cassette YFPi plasmid (Guirimand et al., 2009, 2010). The capacity of interaction of THAS and SGD was characterized by BiFC assays using THAS PCR product cloned via *Spe*I into the pSPYCE(MR) plasmid (Waadt et al., 2008), which allows expression of THAS fused to the carboxy-terminal extremity of the split YFP^C^ fragment (YFP^C^-THAS). The pSCA-SPYNE173-SGD and pSPYNE(R)173-SGD plasmids (Guirimand et al., 2010) were used to express SGD fused to the amino-terminal or carboxy-terminal extremity of the split YFP^N^ fragment (SGD-YFP^N^ and YFP^N^-SGD, respectively). THAS self-interactions were analyzed via additional cloning of the THAS PCR product into the pSCA-SPYNE173 and pSCA-SPYCE(M) plasmids (Guirimand et al., 2010) to express THAS-YFP^N^ and THAS-YFP^C^, respectively. Transient transformation of *C. roseus* cells by particle bombardment and fluorescence imaging were performed following the procedures previously described (Guirimand et al., 2009, 2010).

Complete experimental details are included in the Supplemental Information.

## Author Contributions

A.S. made the initial discovery of THAS activity and conducted all enzyme assays, kinetics, pulldown, and ITC; E.C.T. performed VIGS and assisted in the structural characterization of the enzyme product; E.F. performed the microscopy experiments; L.C. assisted in the purification of THAS and pulldown; F.K. provided initial genomic data that assisted in identification of the THAS candidate; V.C. conceived, initiated, and supervised all localization and BiFC experiments; S.E.O. supervised all enzymology experiments; A.S., V.C., S.E.O. wrote the manuscript.

## Supplemental Information

Document S1. Figures S1–S4 and Supplemental Experimental Procedures

Document S2. Article plus Supplemental Information
