# Supplementary material for: Unlocking the Diversity of Alkaloids in Catharanthus roseus: Nuclear Localization Suggests Metabolic Channeling in Secondary Metabolism
Source: Chem Biol. 2015 Mar 19;22(3):336–41. doi: 10.1016/j.chembiol.2015.02.006 (PMC4372254; doi:10.1016/j.chembiol.2015.02.006)
Supplement: Document S2. Article plus Supplemental Information [file mmc2.pdf]

Brief Communication

Chemistry & Biology

Unlocking the Diversity of Alkaloids in *Catharanthus roseus*: Nuclear Localization Suggests Metabolic Channeling in Secondary Metabolism

Graphical Abstract

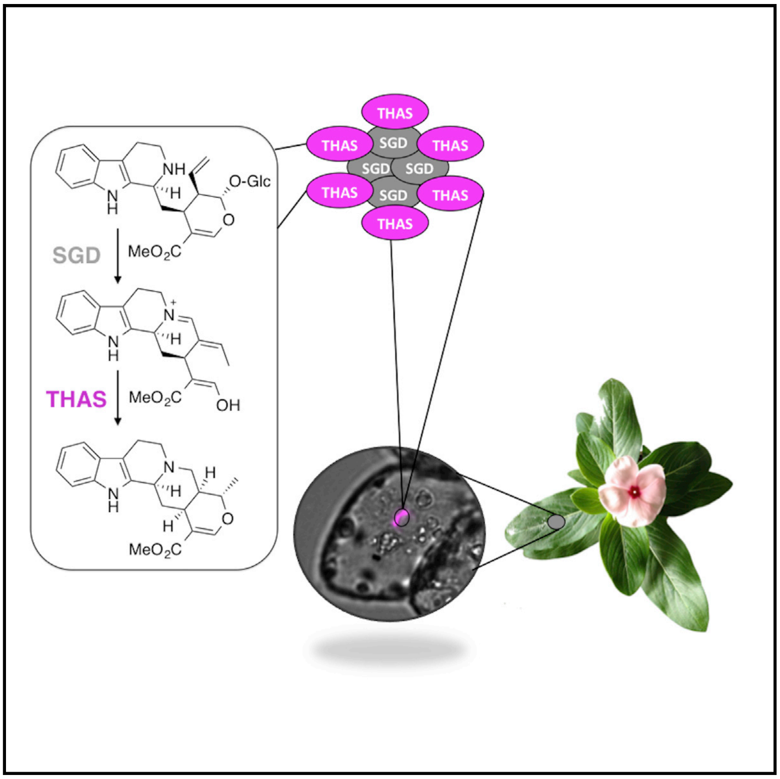

Authors

Anna Stavrinides,
Evangelos C. Tatsis, ...,
Vincent Courdavault,
Sarah E. O'Connor

Correspondence

sarah.oconnor@jic.ac.uk (S.E.O.),
vincent.courdavault@univ-tours.fr (V.C.)

In Brief

How plants transform the central biosynthetic intermediate strictosidine into thousands of divergent alkaloids has remained unresolved. Stavrinides et al. discover a nuclear-localized alcohol dehydrogenase homolog responsible for conversion of strictosidine aglycone to tetrahydroalstonine that appears to interact with an upstream pathway enzyme.

Highlights

- Tetrahydroalstonine synthase catalyzes the formation of a plant-derived alkaloid
- Tetrahydroalstonine synthase is localized to the nucleus
- Tetrahydroalstonine synthase and the preceding pathway enzyme interact
- Discovery of a gene controlling structural diversity of monoterpene indole alkaloids

Stavrinides et al., 2015, Chemistry & Biology *22*, 336–341
March 19, 2015 ©2015 The Authors
http://dx.doi.org/10.1016/j.chembiol.2015.02.006

Chemistry & Biology
Brief Communication

Unlocking the Diversity of Alkaloids in *Catharanthus roseus*: Nuclear Localization Suggests Metabolic Channeling in Secondary Metabolism

Anna Stavrinides,[1] Evangelos C. Tatsis,[1] Emilien Foureau,[2] Lorenzo Caputi,[1] Franziska Kellner,[1] Vincent Courdavault,[2,*] and Sarah E. O'Connor[1,*]
[1]Department of Biological Chemistry, The John Innes Centre, Colney, Norwich NR4 7UH, UK
[2]Université François Rabelais de Tours, EA2106 ''Biomolécules et Biotechnologies Végétales'', 37200 Tours, France
*Correspondence: sarah.oconnor@jic.ac.uk (S.E.O.), vincent.courdavault@univ-tours.fr (V.C.)
http://dx.doi.org/10.1016/j.chembiol.2015.02.006
This is an open access article under the CC BY license (http://creativecommons.org/licenses/by/4.0/).

SUMMARY

The extraordinary chemical diversity of the plant-derived monoterpene indole alkaloids, which include vinblastine, quinine, and strychnine, originates from a single biosynthetic intermediate, strictosidine aglycone. Here we report for the first time the cloning of a biosynthetic gene and characterization of the corresponding enzyme that acts at this crucial branchpoint. This enzyme, an alcohol dehydrogenase homolog, converts strictosidine aglycone to the heteroyohimbine-type alkaloid tetrahydroalstonine. We also demonstrate how this enzyme, which uses a highly reactive substrate, may interact with the upstream enzyme of the pathway.

INTRODUCTION

The monoterpene indole alkaloids (MIAs) are a highly diverse family of natural products that are produced in a wide variety of medicinal plants. Over 3000 members of this natural product class, which includes compounds such as quinine, vinblastine, reserpine, and yohimbine, are derived from a common biosynthetic intermediate, strictosidine aglycone (O'Connor and Maresh, 2006). How plants transform strictosidine aglycone into divergent structural classes has remained unresolved.

The recent availability of transcriptome and genome data has dramatically accelerated the rate at which new plant biosynthetic genes are discovered. All genes that lead to strictosidine aglycone have been recently cloned from the well-characterized medicinal plant *Catharanthus roseus*, which produces over 100 MIAs (De Luca et al., 2014). However, gene products that act on strictosidine aglycone have not been identified in any plant, despite decades of effort. Attempts have been hampered in part by the reactivity and instability of strictosidine aglycone. In *C. roseus*, there are at least two major pathway branches derived from strictosidine aglycone (O'Connor and Maresh, 2006). One pathway is hypothesized to lead to the aspidosperma and the iboga classes to yield the precursors of vinblastine, while the other is expected to lead to alkaloids of the heteroyohimbine type (Figure 1A). These alkaloids have diverse biological activities: vinblastine is used as an anticancer agent (Kaur et al., 2014) and the heteroyohimbines have a range of pharmacological uses (Costa-Campos et al., 1998; Elisabetsky and Costa-Campos, 2006). While it is unknown how many *C. roseus* enzymes use strictosidine aglycone as a substrate, there is clearly more than one enzyme that acts at this crucial branchpoint.

The biochemical pathway leading from strictosidine aglycone to the heteroyohimbine alkaloids has been previously investigated using both crude plant extracts and biomimetic chemistry. Reduction of strictosidine aglycone with $NaBH_4$ or $NaCNBH_3$ yielded the heteroyohimbines ajmalicine (raubasine), tetrahydroalstonine, and 19-epi-ajmalicine, which differ only in the stereochemical configuration at carbons 15, 19, and 20, in various ratios (Figure 1B) (Brown et al., 1977; Kan-Fan and Husson, 1978, 1979, 1980). These three diastereomers were again observed, also in varying relative amounts, when crude *C. roseus* protein extracts were incubated with strictosidine aglycone and NADPH, but not in the absence of NADPH (Rueffer et al., 1979; Stoeckigt et al., 1976, 1977, 1983; Zenk, 1980). Collectively, these observations indicate that the heteroyohimbines result directly from the reduction of strictosidine aglycone and that an NADPH-dependent enzyme is implicated in this process. However, no gene encoding such an enzyme has been identified. Here we report the discovery of a reductase that converts strictosidine aglycone to the heteroyohimbine alkaloid tetrahydroalstonine.

RESULTS AND DISCUSSION

Given that heteroyohimbine biosynthesis likely requires reduction of an iminium present in strictosidine aglycone (Figure 1B), we used a publically available RNA-seq database that we recently generated (Gongora-Castillo et al., 2012) to search for *C. roseus* candidates displaying homology to enzyme classes known to reduce the carbonyl functional group. The alcohol dehydrogenases (ADHs), enzymes that reduce aldehydes and ketones to alcohols, were chosen as the initial focus. As part of a screen of ADHs that are upregulated in response to methyl jasmonate (Gongora-Castillo et al., 2012), a hormone known to upregulate alkaloid biosynthesis, we identified a candidate annotated as sinapyl alcohol dehydrogenase (Supplemental Information). When heterologously expressed and purified from *E. coli* (Figure S1), and assayed with strictosidine aglycone and NADPH, this candidate yielded a product with a mass consistent with a heteroyohimbine (*m/z* 353.1855), thereby implicating this

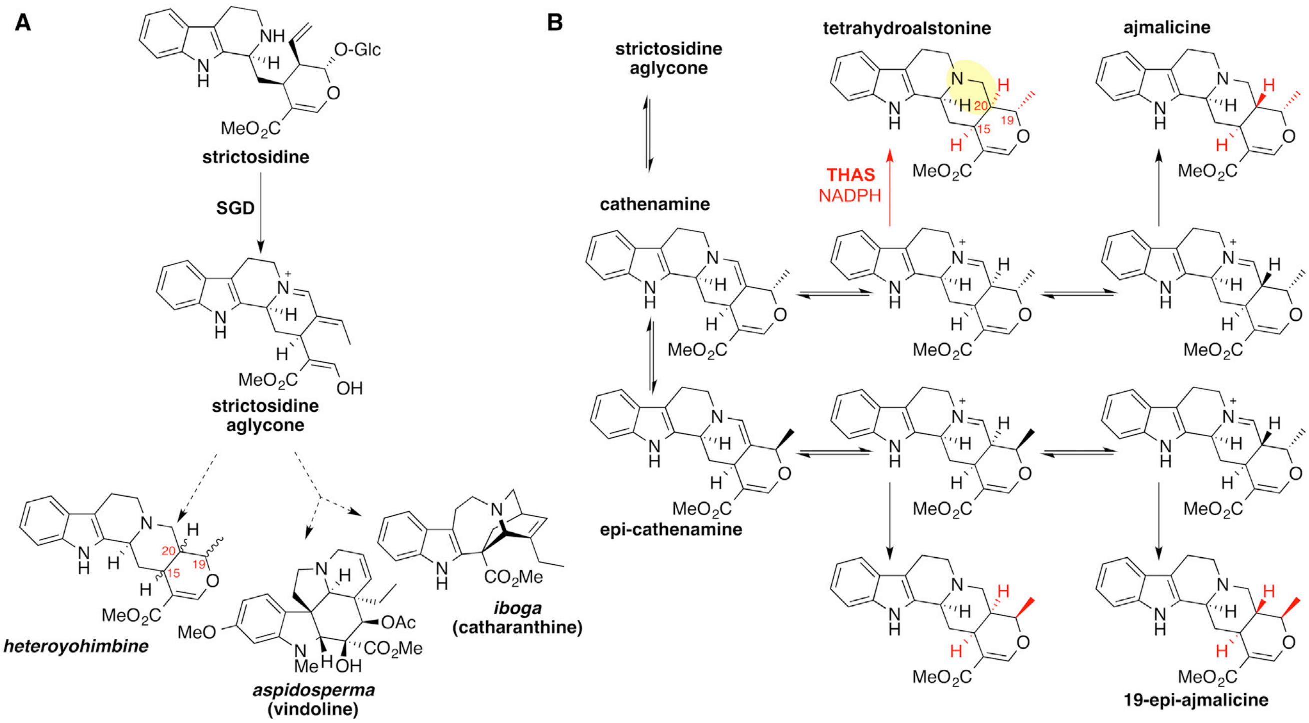

Figure 1. The Monoterpene Indole Alkaloids

(A) Representative monoterpene indole alkaloids derived from strictosidine and strictosidine aglycone found in *Catharanthus roseus*.

(B) Heteroyohimbine biosynthesis.

enzyme in the important structural branchpoint of the MIA biosynthetic pathway (Figure 2A).

To determine the identity of the alkaloid product, the enzyme was incubated with purified strictosidine (4.3 mg) in the presence of strictosidine glucosidase (SGD), which generated strictosidine aglycone in situ to best mimic physiologically relevant conditions. The major product (approximately 1 mg) was isolated by preparative thin-layer chromatography and exhibited an ^{1}H-NMR and ^{13}C-NMR spectrum matching an authentic standard of tetrahydroalstonine (Figure 2B; Figure S2). Hemscheidt and Zenk (1985) previously reported the isolation of an enzyme that produced tetrahydroalstonine, although this protein was purified only 35-fold from *C. roseus* cell cultures. Consistent with Hemscheidt and Zenk's (1985) nomenclature, we named this enzyme tetrahydroalstonine synthase (THAS). A minor enzymatic product was produced in yields too low for NMR characterization, but had a mass and R_f value consistent with ajmalicine, a stereoisomer of tetrahydroalstonine (Figure S2). When applied to normal phase liquid chromatography conditions, ajmalicine and tetrahydroalstonine could be resolved, indicating that the enzyme produces approximately 95% tetrahydroalstonine (Figure 3; Supplemental Information). We also silenced this gene in *C. roseus* seedlings using virus-induced gene silencing (VIGS) (Liscombe and O'Connor, 2011). LC-mass spectrometry (MS) analysis of the silenced leaf tissue showed a statistically significant decrease (approximately 50%) of a peak with a mass and retention time consistent with a heteroyohimbine, suggesting that this enzyme is involved in this biosynthetic pathway branch in vivo (Figure S2). A 50% reduction in product levels upon silencing has been observed for other physiologically relevant biosynthetic genes using the VIGS approach in both *C. roseus* (Asada et al., 2013; Geu-Flores et al., 2012) and another well-studied medicinal plant, opium poppy (Desgagne-Penix and Facchini, 2012; Chen and Facchini, 2014). Therefore, THAS is likely a major producer of tetrahydroalstonine in vivo, although additional, undiscovered *C. roseus* enzymes could also contribute to production of this compound. While we could not resolve tetrahydroalstonine and its stereoisomer ajmalicine in the silenced crude extracts, the levels of the ajmalicine-derived alkaloid serpentine remain the same, suggesting that silencing of THAS does not substantially affect ajmalicine levels and consequently that THAS does not play a major role in the biosynthesis of ajmalicine in planta.

Small-scale assays using LC-MS to monitor product formation indicated that NADPH was required for the reaction, although NADH could also be utilized (Figure S1). Efforts to accurately measure the steady state kinetic constants of this enzyme were complicated because strictosidine aglycone reacts with nucleophiles, opening the possibility that the substrate reacts with components in the reaction or the enzyme. This reactivity has already been associated with a plant defense mechanism involving strictosidine aglycone-mediated aggregation of proteins in *C. roseus* (Guirimand et al., 2010). Nevertheless, we obtained estimated K_m and k_{cat} values (Figure S1). To support these kinetic data, we also performed isothermal titration calorimetry (ITC) with THAS in the presence of NADPH and strictosidine aglycone. Titration of THAS with NADPH indicated that the co-substrate binds first with a K_d of 1.5 ± 0.1 μM (ΔH (cal/mol)

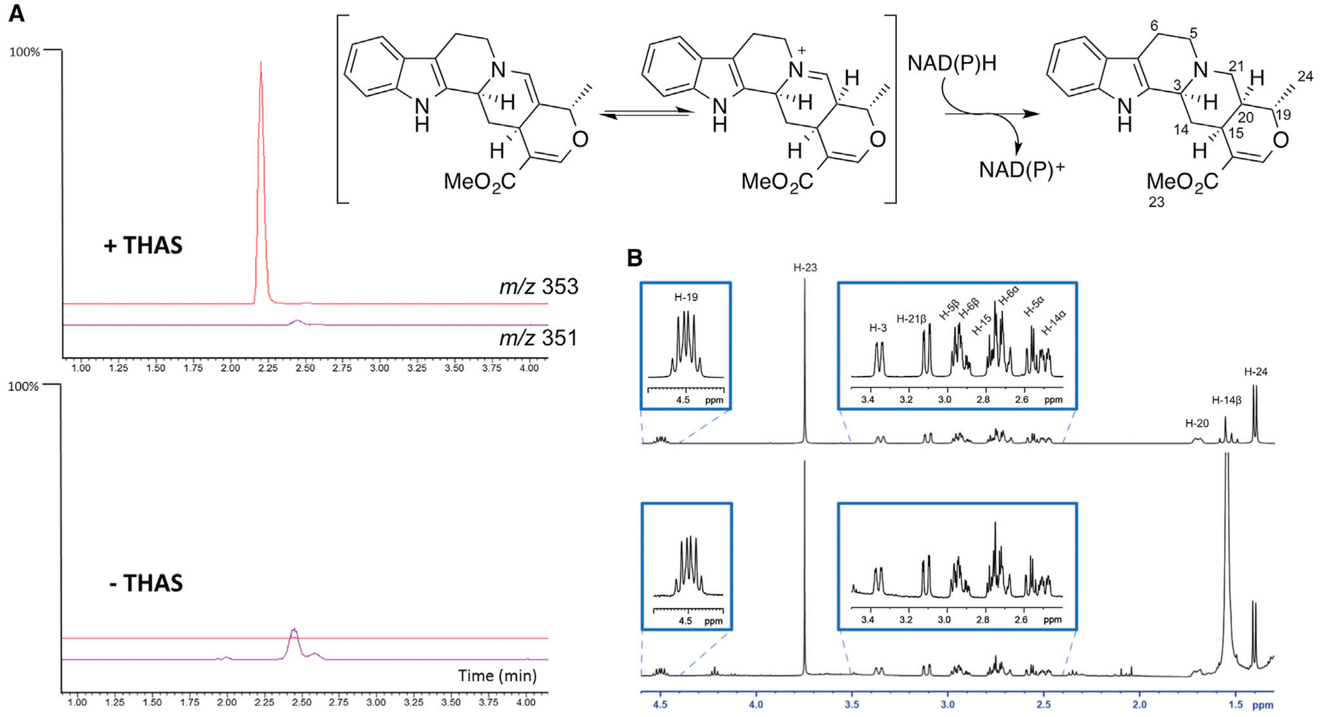

Figure 2. Activity Assays of THAS

Enzyme reactions were performed at 25°C for 30 min and assayed using a mass spectrometer in tandem with ultraperformance liquid chromatography.

(A) The total ion chromatogram for *m/z* 353 (red trace) and *m/z* 351 (purple trace) from 1 to 4 min is shown. Top trace: THAS (50 nM), SGD (6 nM), strictosidine (200 μM), NADPH (200 μM); bottom trace: same reaction in the absence of THAS. The *y* axis represents normalized ion abundance as a percentage relative to $1.00e^8$ detected by selected ion monitoring at *m/z* 353 and 351.

(B) Portion of the ^{1}H-NMR spectrum of the isolated enzymatic product compared with an authentic standard of tetrahydroalstonine.

2310 ± 123.2; Δ*S* (cal/mol/deg) 34.2 ± 0.3) (Figure S1). The aglycone substrate does not appear to bind in the absence of NADPH, suggesting that the enzyme utilizes an ordered binding mechanism in which NADPH binds first. However, titration of the THAS-NADPH complex with strictosidine aglycone led to formation of a precipitate when concentrations of strictosidine aglycone exceeded 60 μM, preventing calculation of an accurate K_d. Collectively, the ITC data for THAS are consistent with an ordered Bi-Bi mechanism, a kinetic mechanism that has been reported for similar ADHs such as cinnamyl alcohol dehydrogenase (Charlier and Plapp, 2000; Lee et al., 2013).

The amino acid sequence of THAS was subjected to a BLAST alignment against the *C. roseus* transcriptome (Gongora-Castillo et al., 2012), as well as the NCBI (Figure S3). The closest characterized homologs of THAS are sinapyl alcohol dehydrogenase (*Populus tremuloides*, 64% amino acid identity), cinnamyl alcohol dehydrogenase (*Populus tomentosa*, 64%) and 8-hydroxygeraniol dehydrogenase (*C. roseus*, 63%), which are zinc-containing medium chain ADHs (Bomati and Noel, 2005; Lee et al., 2013).

Strictosidine aglycone can rearrange into several isomers (Figure 1B), and while it has been reported that the dominant isomer is cathenamine (Gerasimenko et al., 2002; Stoeckigt et al., 1977), equilibration in solution with other isomers occurs (Brown and Leonard, 1979; Stoeckigt et al., 1983). Reduction of cathenamine or epi-cathenamine (Figure 1B) by a reductase would require reduction of the carbon-carbon double bond of an enamine; alternatively, Stoeckigt et al. (1983) and Zenk (1980) suggested that the iminium isomer is reduced (Figure 1B). THAS may catalyze the stereoselective formation of tetrahydroalstonine by selectively binding the correct isomer of the substrate for reduction, thereby relying on the inherent propensity for the enamine and imine to tautomerize under physiological conditions. Given that three diastereomers, ajmalicine, tetrahydroalstonine, and 19-epi-ajmalicine, can be obtained from chemical reduction of strictosidine aglycone, this is a chemically reasonable proposal. An alternative hypothesis is that THAS catalyzes enamine-imine tautomerization in addition to reduction. The difficulties associated with obtaining accurate kinetic data in this system, as well as the inherent reactivity of the strictosidine aglycone, make answering these questions using enzymology approaches challenging. However, identification and comparison with enzymes that generate other heteroyohimbine diastereomers will likely provide the basis for a more definitive mechanism of product specificity.

Recent research has highlighted that plant secondary metabolite biosynthetic pathways often are compartmentalized in different subcellular locations. While microscopy experiments have demonstrated that most of the early steps of monoterpene indole alkaloid biosynthesis in *C. roseus* take place in the cytosol (Courdavault et al., 2014), the enzyme that synthesizes strictosidine is located in the vacuole, and the enzyme SGD, which deglycosylates strictosidine, contains a nuclear localization signal and is in the nucleus, a highly unusual site for secondary

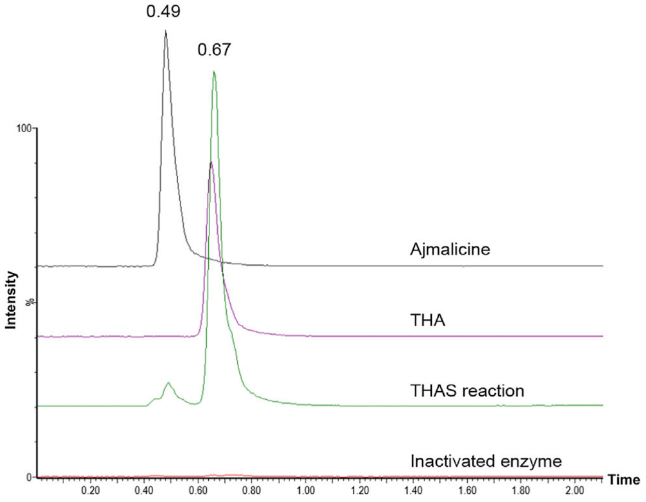

Figure 3. LC-MS Performed under Normal Phase Conditions (Hydrophilic Interaction Liquid Chromatography) Showing Separation of Ajamlicine (Retention Time of 0.49 min) and Tetrahydroalstonine (THA, Retention Time 0.67 min)

THAS produces approximately 95% of the tetrahydroalstonine (THA) diastereomer. The *y* axis represents normalized ion abundance as a percentage detected by selected ion monitoring at *m/z* 353.

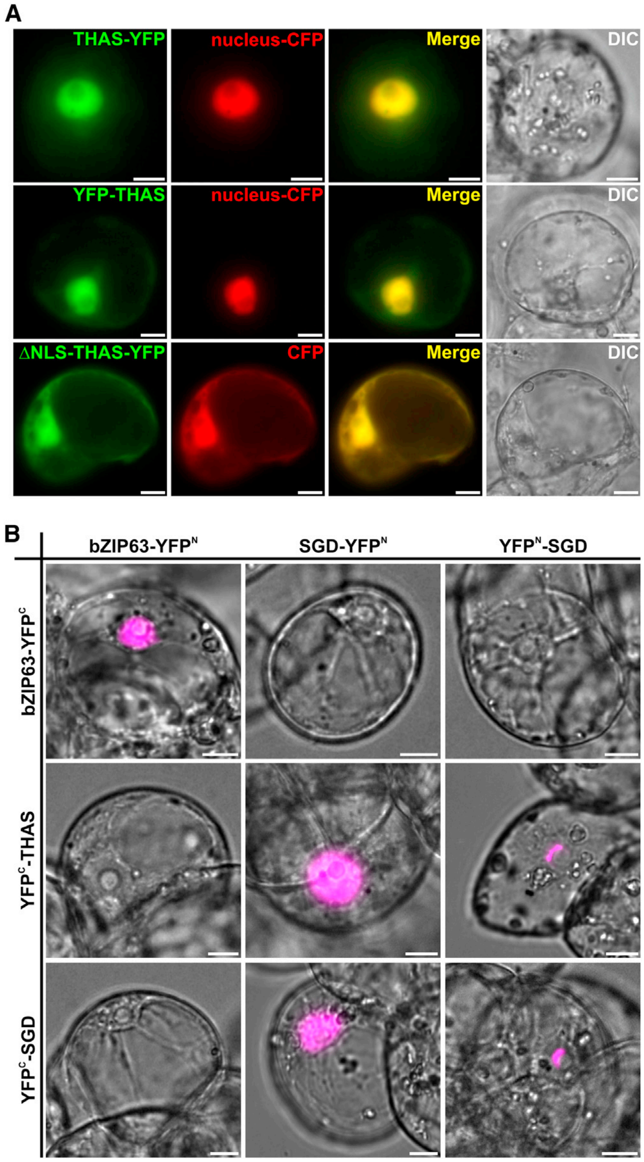

Figure 4. THAS Is Targeted to the Nucleus via a Monopartite Nuclear Localization Signal (NLS) and Interacts with SGD

(A) *C. roseus* cells were transiently cotransformed with plasmids expressing either THAS-YFP (upper row), YFP-THAS (middle row), or the NLS deleted version of THAS (lower row) and plasmids encoding the nuclear CFP marker or the nucleocytosolic CFP marker (second column). Colocalization of the fluorescence signals appears in yellow when merging the two individual (green/red) false color images (third column). Cell morphology is observed with differential interference contrast (DIC) (fourth column).

(B) THAS and SGD interactions were analyzed by BiFC in *C. roseus* cells transiently transformed by plasmids encoding fusions indicated on the top (fusion with the split YFP^N fragment) and on the left (fusion with split YFP^C fragment). bZIP63 was used as a positive BiFC control and to evaluate the specificity of THAS and SGD interactions. The images are merges of the YFP BiFC channel (magenta false color) with the DIC channel to show the nuclear localization of the interactions. Bars, 10 μm.

metabolite biosynthesis (Guirimand et al., 2010). Notably, a motif resembling a class V nuclear localization sequence (Kosugi et al., 2008) was observed in THAS ($K_{214}K_{215}K_{216}R_{217}$). Microscopy of *C. roseus* cells transformed with YFP-tagged THAS confirmed the nuclear location of this enzyme, while deletion of the KKKR sequence disrupted the localization (Figure 4A; Figure S4). This is one of the very few examples of secondary metabolism that is localized to the nucleus (Saslowsky et al., 2005).

Given the reactivity of strictosidine aglycone (Guirimand et al., 2010), metabolic channeling via a protein-protein interaction between SGD and the enzyme immediately downstream may be necessary to protect the substrate. Pull down experiments between SGD and THAS gave partially positive but inconclusive results (Figure S4). However, when we used bimolecular fluorescence complementation (BiFC) in *C. roseus* cells, we observed an interaction between SGD and THAS (Figure 4B). While this interaction generated a diffuse nuclear fluorescent signal when the C-terminal end of SGD was fused to the split-YFP fragment, a sickle-shaped signal was observed when both SGD and THAS were expressed with free C-terminal ends (YFP^N-SGD and YFP^C-THAS). Such a signal was also observed for SGD self-interactions (Guirimand et al., 2010) and likely results from the formation of SGD complexes over 1.5 MDa (Luijendijk et al., 1998). Similar experiments with SGD and an upstream MIA biosynthetic enzyme, loganic acid methyl transferase, failed to show an interaction, highlighting the specificity of this interaction (Figure S4). The fact that THAS interacts with SGD provides further support for the physiological relevance of THAS in planta. As strictosidine aglycone is reactive and most likely toxic in vivo, it has been proposed that this molecule is produced by the plant in response to attack (Guirimand et al., 2010). The nuclear localization of THAS might be an evolutionary mechanism designed to channel this molecule into a more stable product when no such defense is required. Identification of additional nuclear-localized biosynthetic enzymes in *C. roseus* and other heteroyohimbine

producing plants may provide more insight into the reasons for this unusual localization pattern.

SIGNIFICANCE

Many of the monoterpene indole alkaloid structural classes are generated at the SGD junction. Here we report the first identification of a biosynthetic gene that acts directly downstream of SGD. The enzyme, an ADH homolog, generates a heteroyohimbine alkaloid by reducing one of the isomers of strictosidine aglycone. Unusually, this enzyme is located in the nucleus and may interact with its upstream partner, SGD. The discovery of the THAS gene represents the completion of a major branch of monoterpene indole alkaloid biosynthesis, which will now allow reconstruction of heteroyohimbines and heteroyohimbine analogs in heterologous hosts. This discovery is a crucial first step in understanding how the structural diversity of MIAs is controlled.

EXPERIMENTAL PROCEDURES

The THAS gene (accession number KM524258) was cloned into pOPINF and expressed in Rosetta 2 pLysS *E. coli* cells (Novagen) with induction of expression with 0.1 mM isopropyl β-D-1-thiogalactopyranoside. Cultures were grown at 18°C for 16 hr, with shaking at 200 rpm. His-tagged THAS was purified using a HisTrap FF 5-ml column (GE Healthcare). SGD expression and purification was done as described for THAS using the expression system described previously by Yerkes et al. (2008). Purified THAS and SGD were used in all assays. Strictosidine was enzymatically synthesized from tryptamine and a crude methanol extract of snowberries (*Symphoricarpos albus*) enriched in secologanin prepared as previously described (Geerlings et al., 2001). Strictosidine aglycone was generated in situ prior to addition of THAS by incubation of strictosidine and SGD in the appropriate solution for 10 min, at which time strictosidine was completely converted to the aglycone.

Steady state kinetic analyses were performed with 50 nM THAS and 6 nM SGD, 50 mM phosphate buffer (pH 7.5), 200 μM NADPH, and an internal caffeine standard (50 μM). All LC-MS measurements were performed on AQUITY ultra-performance liquid chromatography with a Xevo TQ-S mass spectrometer.

For VIGS, a 330-bp fragment of THAS was cloned into the pTRV2u vector as described (Geu-Flores et al., 2012). The resulting pTRV2u-THAS construct was used to silence THAS in *C. roseus* seedlings essentially as described (Liscombe and O'Connor, 2011).

The subcellular localization of THAS was studied by creating fluorescent fusion proteins using the pSCA-cassette YFPi plasmid (Guirimand et al., 2009, 2010). The capacity of interaction of THAS and SGD was characterized by BiFC assays using THAS PCR product cloned via *Spe*I into the pSPYCE(MR) plasmid (Waadt et al., 2008), which allows expression of THAS fused to the carboxy-terminal extremity of the split YFP^C fragment (YFP^C-THAS). The pSCA-SPYNE173-SGD and pSPYNE(R)173-SGD plasmids (Guirimand et al., 2010) were used to express SGD fused to the amino-terminal or carboxy-terminal extremity of the split YFP^N fragment (SGD-YFP^N and YFP^N-SGD, respectively). THAS self-interactions were analyzed via additional cloning of the THAS PCR product into the pSCA-SPYNE173 and pSCA-SPYCE(M) plasmids (Guirimand et al., 2010) to express THAS-YFP^N and THAS-YFP^C, respectively. Transient transformation of *C. roseus* cells by particle bombardment and fluorescence imaging were performed following the procedures previously described (Guirimand et al., 2009, 2010).

Complete experimental details are included in the Supplemental Information.

SUPPLEMENTAL INFORMATION

Supplemental Information includes Supplemental Experimental Procedures and four figures and can be found with this article online at http://dx.doi.org/10.1016/j.chembiol.2015.02.006.

AUTHOR CONTRIBUTIONS

A.S. made the initial discovery of THAS activity and conducted all enzyme assays, kinetics, pulldown, and ITC; E.C.T. performed VIGS and assisted in the structural characterization of the enzyme product; E.F. performed the microscopy experiments; L.C. assisted in the purification of THAS and pulldown; F.K. provided initial genomic data that assisted in identification of the THAS candidate; V.C. conceived, initiated, and supervised all localization and BiFC experiments; S.E.O. supervised all enzymology experiments; A.S., V.C., S.E.O. wrote the manuscript.

ACKNOWLEDGMENTS

We gratefully acknowledge support from the ERC (311363) and from the Région Centre (France, ABISAL grant). A.S. is supported by a BBSRC DTP studentship.

Received: November 7, 2014
Revised: January 24, 2015
Accepted: February 17, 2015
Published: March 12, 2015

REFERENCES

Asada, K., Salim, V., Masada-Atsumi, S., Edmunds, E., Nagatoshi, M., Terasaka, K., Mizukami, H., and De Luca, V. (2013). A 7-deoxyloganetic acid glucosyltransferase contributes a key step in secologanin biosynthesis in Madagascar periwinkle. Plant Cell *25*, 4123–4134.

Bomati, E.K., and Noel, J.P. (2005). Structural and kinetic basis for substrate selectivity in *Populus tremuloides* sinapyl alcohol dehydrogenase. Plant Cell *17*, 1598–1611.

Brown, R.T., and Leonard, J. (1979). Biomimetic synthesis of cathenamine and 19-epicathenamine, key intermediates to heteroyohimbine alkaloids. J. Chem. Soc. Chem. Commun. 877–879.

Brown, R.T., Leonard, J., and Sleigh, S.K. (1977). 'One-pot' biomimetic synthesis of 19β-heteroyohimbine alkaloids. J. Chem. Soc. Chem. Commun. 636–638.

Charlier, H.A., and Plapp, B.V. (2000). Kinetic cooperativity of human liver alcohol dehydrogenase γ2. J. Biol. Chem. *275*, 11569–11575.

Chen, X., and Facchini, P.J. (2014). Short-chain dehydrogenase/reductase catalyzing the final step of noscapine biosynthesis is localized to laticifers in opium poppy. Plant J. *77*, 173–184.

Costa-Campos, L., Lara, D.R., Nunes, D.S., and Elisabetsky, E. (1998). Antipsychotic-like profile of alstonine. Pharmacol. Biochem. Behav. *60*, 133–141.

Courdavault, V., Papon, N., Clastre, M., Giglioli-Guivarc'h, N., St-Pierre, B., and Burlat, V. (2014). A look inside an alkaloid multisite plant: the *Catharanthus* logistics. Curr. Opin. Plant Biol. *19*, 43–50.

De Luca, V., Salim, V., Thamm, A., Masada, S.A., and Yu, F. (2014). Making iridoids/secoiridoids and monoterpenoid indole alkaloids: progress on pathway elucidation. Curr. Opin. Plant Biol. *19*, 35–42.

Desgagne-Penix, I., and Facchini, P.J. (2012). Systematic silencing of benzylisoquinoline alkaloid biosynthetic genes reveals the major route to papaverine in opium poppy. Plant J. *72*, 331–344.

Elisabetsky, E., and Costa-Campos, L. (2006). The alkaloid alstonine: a review of its pharmacological properties. Evidence-based complementary and alternative medicine. Evid. Based Complement. Alternat. Med. *3*, 39–48.

Geerlings, A., Redondo, F.J., Contin, A., Memelink, J., van der Heijden, R., and Verpoorte, R. (2001). Biotransformation of tryptamine and secologanin into plant terpenoid indole alkaloids by transgenic yeast. Appl. Microbiol. Biotechnol. *56*, 420–424.

Gerasimenko, I., Sheludko, Y., Ma, X., and Stockigt, J. (2002). Heterologous expression of a *Rauvolfia* cDNA encoding strictosidine glucosidase, a biosynthetic key to over 2000 monoterpenoid indole alkaloids. Eur. J. Biochem. *269*, 2204–2213.

Geu-Flores, F., Sherden, N.H., Courdavault, V., Burlat, V., Glenn, W.S., Wu, C., Nims, E., Cui, Y., and O'Connor, S.E. (2012). An alternative route to cyclic terpenes by reductive cyclization in iridoid biosynthesis. Nature *492*, 138–142.

Gongora-Castillo, E., Childs, K.L., Fedewa, G., Hamilton, J.P., Liscombe, D.K., Magallanes-Lundback, M., Mandadi, K.K., Nims, E., Runguphan, W., Vaillancourt, B., et al. (2012). Development of transcriptomic resources for interrogating the biosynthesis of monoterpene indole alkaloids in medicinal plant species. PLoS One *7*, e52506.

Guirimand, G., Burlat, V., Oudin, A., Lanoue, A., St-Pierre, B., and Courdavault, V. (2009). Optimization of the transient transformation of *Catharanthus roseus* cells by particle bombardment and its application to the subcellular localization of hydroxymethylbutenyl 4-diphosphate synthase and geraniol 10-hydroxylase. Plant Cell Rep. *28*, 1215–1234.

Guirimand, G., Courdavault, V., Lanoue, A., Mahroug, S., Guihur, A., Blanc, N., Giglioli-Guivarc'h, N., St-Pierre, B., and Burlat, V. (2010). Strictosidine activation in Apocynaceae: towards a "nuclear time bomb"? BMC Plant Biol. *10*, 182.

Hemscheidt, T., and Zenk, M.H. (1985). Partial purification and characterization of a NADPH dependent tetrahydroalstonine synthase from *Catharanthus roseus* cell suspension cultures. Plant Cell Rep. *4*, 216–219.

Kan-Fan, C., and Husson, H.P. (1978). Stereochemical control in the biomimetic conversion of heteroyohimbine alkaloid precursors. Isolation of a novel key intermediate. J. Chem. Soc. Chem. Commun. 618–619.

Kan-Fan, C., and Husson, H.P. (1979). Isolation and biomimetic conversion of 4,21-dehydrogeissoschizine. J. Chem. Soc. Chem. Commun. 1015–1016.

Kan-Fan, C., and Husson, H.P. (1980). Biomimetic synthesis of yohimbine and heteroyohimbine alkaloids from 4,21-dehydrogeissoschizine. Tetrahedron Lett. *21*, 1463–1466.

Kaur, R., Kaur, G., Gill, R.K., Soni, R., and Bariwal, J. (2014). Recent developments in tubulin polymerization inhibitors: an overview. Eur. J. Med. Chem. *87C*, 89–124.

Kosugi, S., Hasebe, M., Matsumura, N., Takashima, H., Miyamoto-Sato, E., Miya, E., Tomita, M., and Yanagawa, H. (2008). Six classes of nuclear localization signals specific to different binding grooves of importin alpha. J. Biol. Chem. *284*, 478–485.

Lee, C., Bedgar, D.L., Davin, L.B., and Lewis, N.G. (2013). Assessment of a putative proton relay in *Arabidopsis* cinnamyl alcohol dehydrogenase catalysis. Org. Biomol. Chem. *11*, 1127–1134.

Liscombe, D.K., and O'Connor, S.E. (2011). A virus-induced gene silencing approach to understanding alkaloid metabolism in *Catharanthus roseus*. Phytochemistry *72*, 1969–1977.

Luijendijk, T.J.C., Stevens, L.H., and Verpoorte, R. (1998). Purification and characterisation of strictosidine β-D-glucosidase from *Catharanthus roseus* cell suspension cultures. Plant Physiol. Biochem. *36*, 419–425.

O'Connor, S.E., and Maresh, J.J. (2006). Chemistry and biology of monoterpene indole alkaloid biosynthesis. Nat. Prod. Rep. *23*, 532–547.

Rueffer, M., Kan-Fan, C., Husson, H.P., Stoeckigt, J., and Zenk, M.H. (1979). 4,21-Dehydrogeissoschizine, an intermediate in heteroyohimbine alkaloid biosynthesis. J. Chem. Soc. Chem. Commun. 1016–1018.

Saslowsky, D.E., Warek, U., and Winkel, B.S. (2005). Nuclear localization of flavonoid enzymes in *Arabidopsis*. J. Biol. Chem. *280*, 23235–23740.

Stoeckigt, J., Treimer, J., and Zenk, M.H. (1976). Synthesis of ajmalicine and related indole alkaloids by cell free extracts of *Catharanthus roseus* cell suspension cultures. FEBS Lett. *70*, 267–270.

Stoeckigt, J., Husson, H.P., Kan-Fan, C., and Zenk, M.H. (1977). Cathenamine, a central intermediate in the cell free biosynthesis of ajmalicine and related indole alkaloids. J. Chem. Soc. Chem. Commun. 164–166.

Stoeckigt, J., Hemscheidt, T., Hoefle, G., Heinstein, P., and Formacek, V. (1983). Steric course of hydrogen transfer during enzymatic formation of 3α-heteroyohimbine alkaloids. Biochemistry *22*, 3448–3452.

Waadt, R., Schmidt, L.K., Lohse, M., Hashimoto, K., Bock, R., and Kudla, J. (2008). Multicolor bimolecular fluorescence complementation reveals simultaneous formation of alternative CBL/CIPK complexes in planta. Plant J. *56*, 505–516.

Yerkes, N., Wu, J.X., McCoy, E., Galan, M.C., Chen, S., and O'Connor, S.E. (2008). Substrate specificity and diastereoselectivity of strictosidine glucosidase, a key enzyme in monoterpene indole alkaloid biosynthesis. Bioorg. Med. Chem. Lett. *18*, 3095–3098.

Zenk, M.H. (1980). Enzymic synthesis of ajmalicine and related indole alkaloids. J. Nat. Prod. *43*, 438–451.

Chemistry & Biology, Volume *22*

Supplemental Information

Unlocking the Diversity of Alkaloids in *Catharanthus roseus*: Nuclear Localization Suggests Metabolic Channeling in Secondary Metabolism

Anna Stavrinides, Evangelos C. Tatsis, Emilien Foureau, Lorenzo Caputi, Franziska Kellner, Vincent Courdavault, and Sarah E. O'Connor

Supplemental Information for

Unlocking the diversity of alkaloids in *Catharanthus roseus*: nuclear localization suggests metabolic channeling in secondary metabolism

Anna Stavrinides[1], Evangelos C. Tatsis[1], Emilien Foureau[2], Lorenzo Caputi[1], Franziska Kellner[1], Vincent Courdavault[2]*, Sarah E. O'Connor[1]*

1. The John Innes Centre, Department of Biological Chemistry, Colney, Norwich NR4 7UH, UK

2. Université François Rabelais de Tours, EA2106 "Biomolécules et Biotechnologies Végétales"; 37200 Tours, France

vincent.courdavault@univ-tours.fr; sarah.oconnor@jic.ac.uk

Supplemental Data: Figures

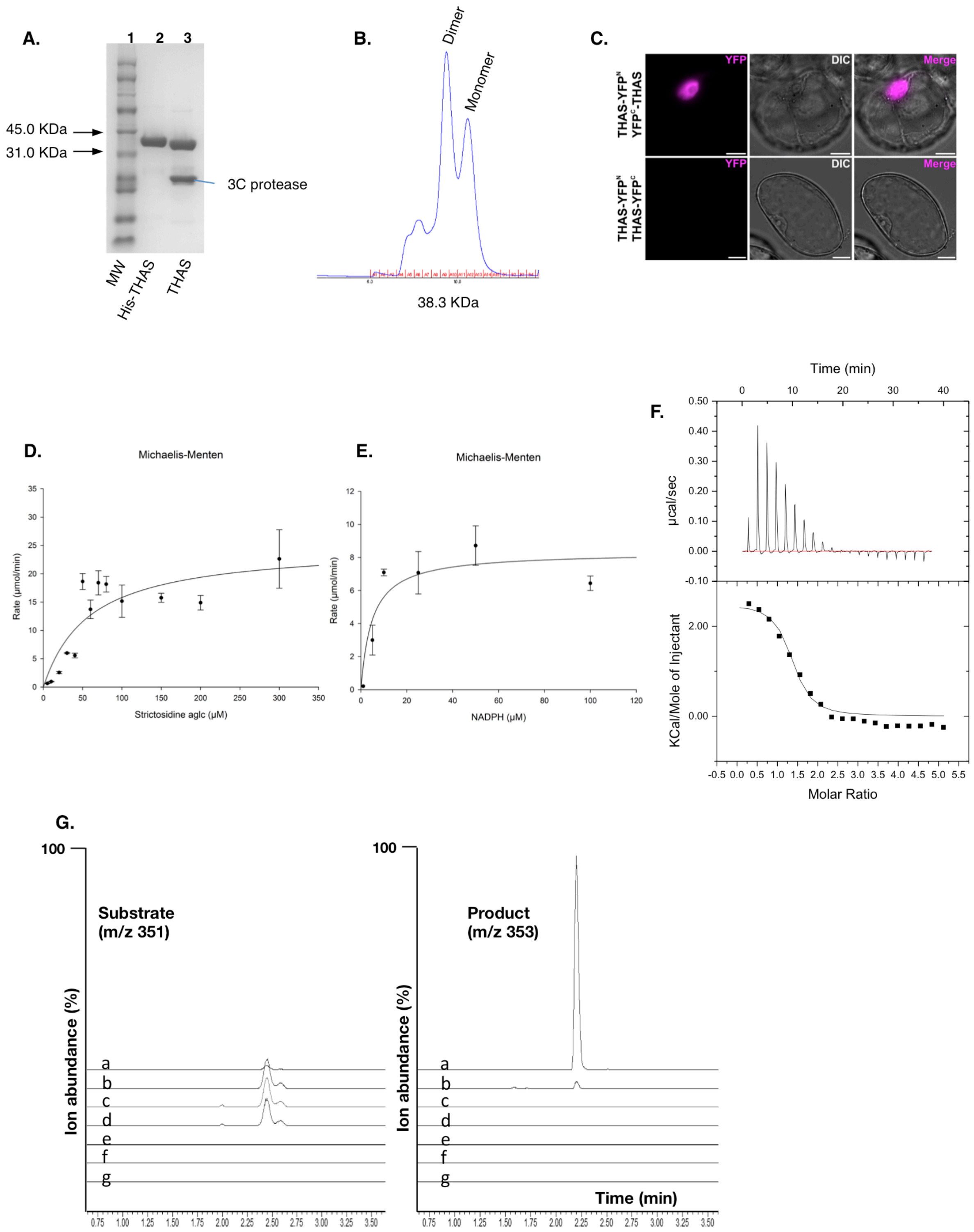
A.
1 2 3
45.0 KDa
31.0 KDa
3C protease
MW
His-THAS
THAS
B.
Dimer
Monomer
38.3 KDa
C.
THAS-YFPᴺ YFPᶜ-THAS
THAS-YFPᴺ THAS-YFPᶜ
YFP
DIC
Merge
D.
Michaelis-Menten
Rate (μmol/min)
Strictosidine aglc (μM)
E.
Michaelis-Menten
Rate (μmol/min)
NADPH (μM)
F.
Time (min)
μcal/sec
KCal/Mole of Injectant
Molar Ratio
G.
Substrate (m/z 351)
Product (m/z 353)
Ion abundance (%)
Time (min)
a
b
c
d
e
f
g

Supplemental Figure S1, related to Figure 1**.** Characterization of THAS.
A. Purification of THAS on ÅKTA using Ni-NTA followed by gel filtration chromatography. The 6X-His tag could be cleaved by 3C protease.
B. Gel filtration chromatogram showing monomer and dimer states of THAS.
C. THAS homodimerization was confirmed by BiFC in *C. roseus* cells transiently transformed by a combination of plasmids encoding THAS-YFP^N and YFP^C-THAS (upper row) or THAS-YFP^N and THAS-YFP^C (lower row). The presented images are merges of the YFP BiFC channel (magenta false color) with the DIC channel to show the nuclear localization of the interactions. While no interaction can be observed for THAS-YFP^N and THAS-YFP^C (lower row), formation of BiFC complexes for THAS-YFP^N and YFP^C-THAS strongly suggests that THAS undergoes a head-to-tail homodimerization. Bars 10 μm.
D. Michaelis-Menten steady state kinetics of THAS for strictosidine aglycone at saturating NADPH concentration (200 μM). The large errors, which are likely the result of the reactivity of the strictosidine aglycone substrate, prevent more detailed kinetic analysis.
E. Exponential curve for NADPH at saturating strictosidine concentration (300 μM). Approximate kinetic constants: K_m (strictosidine aglycone) 58.29 ± 20.7 μM; K_m (NADPH) 4.84 ± 2.5 μM; k_{cat} 8.34 s^{-1}.
F. Isothermal titration calorimetry (ITC) for NADPH binding to THAS. Titration of THAS with NADPH indicates that the cofactor binds first with a K_d of 1.5 ± 0.1 µM (ΔH (cal/mol) 2310 ± 123.2; ΔS (cal/mol/deg) 34.2 ± 0.3), which is consistent with reactions catalyzed by similar alcohol dehydrogenases.
G. Activity assays of THAS. Enzyme reactions were performed at 25˚C for 30 minutes. Total ion chromatogram for *m/z* 353, y axis represents ion abundance as a percentage relative to 1.00e^8. Trace a: THAS (50 nM), SGD (6 nM), strictosidine (200 µM), NADPH (200 µM); b: NADPH replaced with NADH; c: boiled THAS; d: no THAS; e: no strictosidine; f: no SGD; g: no SGD, no THAS.

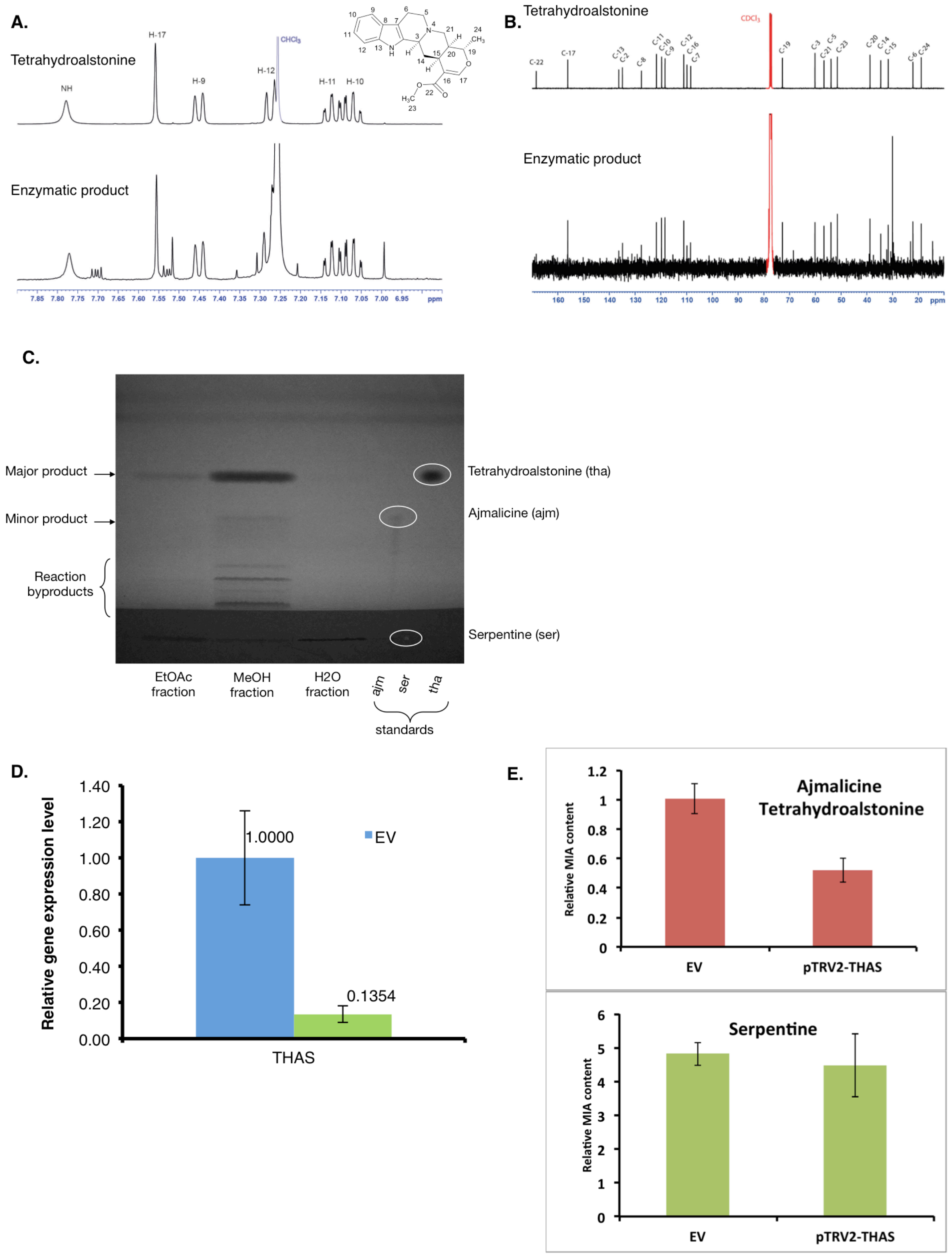
A.
Tetrahydroalstonine
Enzymatic product
NH
H-17
H-9
H-12
CHCl3
H-11
H-10
ppm
B.
Tetrahydroalstonine
Enzymatic product
CDCl3
C-22
C-17
C-19
ppm
C.
Major product
Minor product
Reaction byproducts
Tetrahydroalstonine (tha)
Ajmalicine (ajm)
Serpentine (ser)
EtOAc fraction
MeOH fraction
H2O fraction
ajm
ser
tha
standards
D.
Relative gene expression level
1.0000
0.1354
EV
THAS
E.
Relative MIA content
Ajmalicine
Tetrahydroalstonine
EV
pTRV2-THAS
Serpentine

Supplemental Figure S2, related to Figure 2**.** Characterization of THAS product.
A. ^{1}H NMR Comparison of the major enzymatic product (bottom trace) with authentic standard of tetrahydroalstonine (top trace), aromatic region.
B. Characterization of the major product by ^{13}C NMR.
C. Large-scale production for NMR characterisation isolated by preparative thin layer chromatography using UV detection. Reaction by products are likely decomposition products of strictosidine aglycone.
D. Real time PCR showing downregulation of THAS in silenced (VIGS) leaves. Relative gene expression level of THAS in *C. roseus* leaves that have been inoculated with pTRV2 Empty vector (EV) and pTRV2-THAS. The decrease of THAS gene expression due to VIGS is ≈ 85% compared to EV.
E. Mass spectrometry profiles of silenced and empty vector control leaves that show statistically significant decrease in heteroyohimbine levels (P value 0.00199). The two diastereomers, ajmalicine and tetrahydroalstonine, cannot be separated under a variety of conditions on LCMS (see Supplemental Results). A peak corresponding to serpentine (*m/z* 349), which is derived from ajmalicine, does not significantly decrease in response to silencing of THAS. While these data indicate that THAS is involved in tetrahydroalstonine biosynthesis *in vivo*, we do not rule out the existence of additional *C. roseus* enzymes that catalyze tetrahydroalstonine formation.

A.

```
Score = 11670
Length of alignment = 361
Sequence  SAD :  1 - 362 (Sequence length = 362)
Sequence THAS :  1 - 356 (Sequence length = 356)

 SAD MSKSPEEE-HPVKAFGWAARDQSGHLSPFNFSRRATGEEDVRFKVLYCGVCHSDLHSIKNDWGFSMY
      |||| ||  |||||| ||.| || .||||||||||||.||..|||||| |. | .  ||  ||. |
THAS ASKSPSEEVYPVKAFGLAAKDSSGLFSPFNFSRRATGEHDVQLKVLYCGTCQYDREMSKNKFGFTSY

 SAD PLVPGHEIVGEVTEVGSKVKKVNVGDKVGVGCLVGACHSCESCANDLENYCPKMILTYASIYHDGTI
     | | ||||||||||||||.| .|||||||. .. .|  || |.|..|||||      |.
THAS PYVLGHEIVGEVTEVGSKVQKFKVGDKVGVASIIETCGKCEMCTNEVENYCPE-----AGSI---DS

 SAD TYGGYSDHMVANERYIIRFPDNMPLDGGAPLLCAGITVYSPLKYFGLDEPGKHIGIVGLGGLGHVAV
      ||. |.  | || ..|| |.|.|||.| |||||||| |||.| .||| |||.||| |||||||||.
THAS NYGACSNIAVINENFVIRWPENLPLDSGVPLLCAGITAYSPMKRYGLDKPGKRIGIAGLGGLGHVAL

 SAD KFAKAFGSKVTVISTSPSKKEEALKNFGADSFLVSRDQEQMQAAAGTLDGIIDTVSAVHPLLPLFGL
     .||||||.||||||.|  || ||. .|||||||||  . |.||.|||||||||||... |.| ||..|
THAS RFAKAFGAKVTVISSSLKKKREAFEKFGADSFLVSSNPEEMQGAAGTLDGIIDTIPGNHSLEPLLAL

 SAD LKSHGKLILVGAPEKPLELPAFSLIAGRKIVAGSGIGGMKETQEMIDFAAKHNITADIEVISTDYLN
     ||. ||||..|||| |.|.|| ||. | |..|.|  |.||| ||||.||| ||| ||.|||| ||.|
THAS LKPLGKLIILGAPEMPFEVPAPSLLMGGKVMAASTAGSMKEIQEMIEFAAEHNIVADVEVISIDYVN

 SAD TAMERLAKNDVRYRFVIDVGNTLAAT
     |||||| ..|||||||||.|||| .
THAS TAMERLDNSDVRYRFVIDIGNTLKSN

Percentage ID = 63.99
```

B.

Accession	E value	Coverage	Sequence Identity
cra_locus_17172_iso_2_len_1238_ver_3	1.1e-149	53.19%	75.98%
cra_locus_17172_iso_1_len_1081_ver_3	8.4e-121	50.53%	77.32%
cra_locus_54575_iso_1_len_1197_ver_3	1.5e-116	93.09%	62.29%
cra_locus_3755_iso_6_len_1410_ver_3	1.1e-115	94.95%	61.10%
cra_locus_3755_iso_4_len_1300_ver_3	3.4e-104	88.56%	58.94%
cra_locus_3755_iso_1_len_1256_ver_3	7.4e-100	63.03%	61.22%
cra_locus_3755_iso_2_len_1439_ver_3	2.0e-93	75.53%	61.99%
cra_locus_2876_iso_6_len_1144_ver_3	1.1e-88	76.33%	60.00%
cra_locus_4105_iso_5_len_1418_ver_3	4.6e-87	63.03%	69.29%
cra_locus_4105_iso_2_len_1516_ver_3	2.8e-85	43.62%	64.88%
cra_locus_426_iso_1_len_1300_ver_3	1.4e-84	77.13%	56.04%
cra_locus_426_iso_5_len_1300_ver_3	1.8e-84	76.33%	56.61%
cra_locus_4105_iso_1_len_1400_ver_3	5.7e-82	63.03%	67.63%
cra_locus_6243_iso_9_len_810_ver_3	1.7e-79	55.85%	72.04%
cra_locus_426_iso_2_len_1230_ver_3	6.9e-78	71.01%	56.00%
cra_locus_6243_iso_4_len_951_ver_3	1.8e-75	38.56%	71.72%

Supplemental Figure S3, related to Figure 3**.** Sequence similarity of THAS with other enzymes.

A. Pairwise protein alignment of THAS with sinapyl alcohol dehydrogenase (SAD; pdb 1YQD), the nearest functionally characterized protein homolog (64% amino acid identity); green: catalytic residues; light blue: catalytic Zn^{2+} ligand sphere; dark blue: structural Zn ligand sphere; gray: NADP(H) binding; red: nuclear localization signal.

B. The top hits of a tBLASTn search of the THAS amino acid sequence against the *C. roseus* transcriptome. The function of these transcripts has not been reported. Transcripts highlighted in gray have negligible expression levels in elicited seedlings (< 30 FPKM; THAS (locus_1974) is > 500 FPKM). The transcriptome and expression values can be downloaded from http://medicinalplantgenomics.msu.edu. The tBLASTn search was also performed at the medicinal plant genomics website.

.

A.

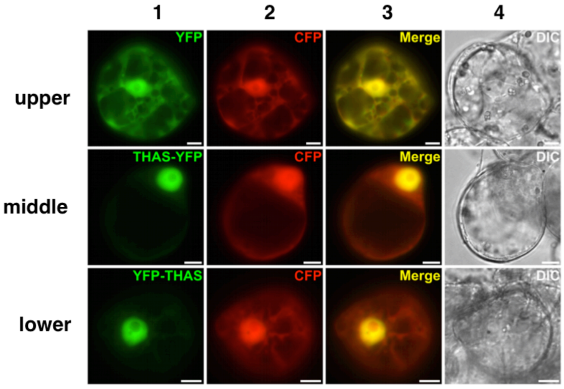
1
2
3
4
upper
middle
lower
YFP
CFP
Merge
DIC
THAS-YFP
YFP-THAS

B.

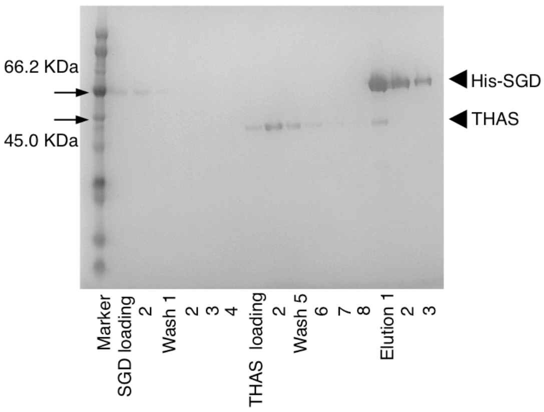
66.2 KDa
45.0 KDa
His-SGD
THAS
Marker
SGD loading
2
Wash 1
2
3
4
THAS loading
2
Wash 5
6
7
8
Elution 1
2
3

C.

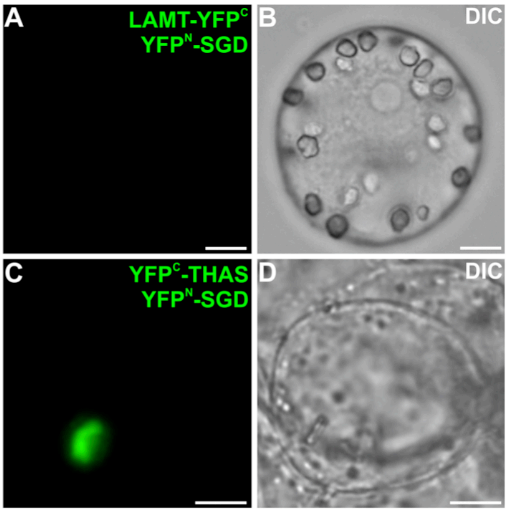
A
LAMT-YFP^{C}
YFP^{N}-SGD
B
DIC
C
YFP^{C}-THAS
YFP^{N}-SGD
D
DIC

Supplemental Figure S4, related to Figure 4**.** Localization of THAS. **A.** THAS is targeted to the nucleus via a monopartite NLS. *C. roseus* cells were transiently co-transformed with plasmids expressing free YFP (upper row), THAS-YFP (middle row) or YFP-THAS (lower row) and plasmid encoding the nucleocytosolic CFP marker (2nd column). Co-localization of the fluorescence signals appears in yellow when merging the two individual (green/red) false-color images (3rd column). Cell morphology is observed with differential interference contrast (DIC) (4th column). Free YFP exhibits a typical nucleocytosolic localization while both THAS-YFP and YFP-THAS are primarily targeted to the nucleus with a residual cytosolic localization barely detectable. *Bars* 10 μm. **B.** Pull down of THAS with strictosidine glucosidase (SGD) as bait. His-SGD (380 μg) loaded onto His-column (V=1mL) followed by a wash with buffer (5 mL total). THAS (100 μg), preloaded with NADPH (0.5 mM), was then loaded (V=1mL). Elution was performed with 250mM imidazole washes (3 mL total). **C.** A negative control with an upstream biosynthetic enzyme LAMT (loganic acid methyl transferase) to demonstrate the specificity of the THAS/SGD interaction. LAMT/SGD (A) and THAS/SGD (C) interactions were analyzed by BiFC in *C. roseus* cells transiently transformed by plasmids encoding the indicated fusions. Cell morphology (B and D) is observed with differential interference contrast (DIC). No interaction with LAMT and SGD is observed. *Bars* 10 μm.

Supplemental Experimental Procedures

A. Cloning of THAS

The gene coding THAS was amplified from *C. roseus* leaf cDNA using primers designed based on the sequence from the expression reads (http://medicinalplantgenomics.msu.edu). The open reading frame of the gene was amplified using the primer pair 5'-AAGTTCTGTTTCAGGGCCCG*GCAATGGCTTCAAA*-3' and 5'-ATGGTCTAGAAAGCTTTA*ATTTGATTTCAGAGTGTTC*-3' (gene specific sequence in italics), and cloned into the *E. coli* expression vector pOPINF (Berrow et al., 2007) using the In-fusion cloning system (Takara Clontech). The identity of the sequence was confirmed by sequencing (Source Bioscience). The expression levels of THAS and SGD in different *C. roseus* tissues are shown below (http://medicinalplantgenomics.msu.edu).

Locus	Enzyme	seedlings	seedlings elicited 5 days	seedlings elicited 12 days	Flowers	mature leaf	immature leaf	stem	root
1974_iso_3	THAS	26.20	505.76	551.69	2.62	17.33	20.19	114.48	13.41
2046_iso_8	SGD	153.33	862.09	407.86	13.02	40.75	73.06	85.47	51.65

THAS cDNA sequence (1071 bp):

ATGGCAATGGCTTCAAAGTCACCTTCTGAAGAAGTATATCCAGTGAAGGCATTTG
GTTTGGCTGCTAAGGATTCTTCTGGGCTTTTCTCTCCATTCAACTTCTCAAGAAG
GGCCACAGGGGAACACGATGTGCAGCTCAAAGTATTATACTGTGGGACTTGCCA
ATATGACAGGGAAATGAGCAAAAACAAATTTGGATTTACAAGCTATCCTTATGTTT
TAGGGCATGAAATTGTGGGTGAGGTAACTGAAGTTGGCAGCAAGGTGCAGAAAT
TCAAAGTCGGGGACAAAGTGGGCGTAGCAAGCATAATTGAAACTTGTGGCAAAT
GTGAAATGTGTACAAATGAAGTTGAAAATTACTGTCCAGAAGCAGGATCAATAGA
CAGCAATTACGGGGCATGTTCAAATATAGCAGTGATAAACGAGAATTTTGTCATC

CGTTGGCCTGAAAATCTTCCTTTGGATTCTGGTGTTCCTCTTCTATGTGCAGGAA
TCACGGCTTATAGTCCCATGAAACGTTATGGACTTGATAAACCTGGAAAACGTAT
CGGCATAGCCGGTCTAGGAGGACTTGGACATGTAGCTCTTAGATTTGCCAAAGC
TTTTGGGGCTAAGGTGACAGTGATTAGTTCTTCACTTAAGAAAAAACGTGAAGCC
TTTGAGAAATTCGGAGCAGATTCTTTCTTGGTCAGCAGTAATCCAGAAGAAATGC
AGGGTGCAGCAGGAACATTGGATGGGATCATAGACACTATACCAGGGAATCACT
CTCTTGAGCCACTCCTTGCTTTATTGAAGCCTCTTGGGAAGCTTATCATTTTAGG
TGCACCAGAAATGCCCTTTGAGGTTCCCGCTCCTTCCCTGCTTATGGGTGGAAA
AGTAATGGCTGCCAGTACTGCTGGGAGTATGAAGGAAATACAAGAGATGATTGA
ATTTGCAGCAGAACACAACATAGTAGCAGATGTGGAGGTTATCTCTATTGACTAT
GTGAACACTGCAATGGAGCGCCTTGATAACTCTGATGTGAGATATCGTTTCGTG
ATTGATATAGGGAACACTCTGAAATCAAAT**TAA**

ADH4 protein sequence (356 aa):

MAMASKSPSEEVYPVKAFGLAAKDSSGLFSPFNFSRRATGEHDVQLKVLYCGTCQ
YDREMSKNKFGFTSYPYVLGHEIVGEVTEVGSKVQKFKVGDKVGVASIIETCGKCE
MCTNEVENYCPEAGSIDSNYGACSNIAVINENFVIRWPENLPLDSGVPLLCAGITAYS
PMKRYGLDKPGKRIGIAGLGGLGHVALRFAKAFGAKVTVISSSLKKKREAFEKFGAD
SFLVSSNPEEMQGAAGTLDGIIDTIPGNHSLEPLLALLKPLGKLIILGAPEMPFEVPAP
SLLMGGKVMAASTAGSMKEIQEMIEFAAEHNIVADVEVISIDYVNTAMERLDNSDVR
YRFVIDIGNTLKSN

B. Enzyme expression and purification

The THAS gene was expressed in Rosetta 2 pLysS *E. coli* cells (Novagen®, Merck Millipore, Massachusetts, USA). A starter culture was grown overnight at 37˚C in 20

mL of LB media supplemented with carbenicillin and chloramphenicol (100 µg/mL and 34 µg/mL respectively). A 1:100 dilution in fresh LB media supplemented with antibiotics was prepared and allowed to grow at 37˚C to an OD_{600} of 0.6. Before induction of expression with 0.1 mM IPTG, the cultures were cooled to 18˚C and kept at this temperature until harvest, with 200 rpm shaking. After 16 h the cells were collected by centrifugation and resuspended in 50 mL Buffer A (50 mM Tris-HCl pH 8, 50 mM glycine, 500 mM NaCl, 5% glycerol, 20 mM imidazole) along with a tablet of protease inhibitor (Roche Diagnostics Ltd.). Cells were lysed using sonication for 3 minutes on ice using 2 s pulses. All purification steps were performed at 4˚C on an ÄKTAxpress purifier (GE Healthcare). His-tagged THAS was purified using a HisTrap FF 5 mL column (GE Healthcare) equilibrated with Buffer A. The sample was loaded at a flow rate of 4 mL/min and step-eluted with Buffer B (50 mM Tris-HCl pH 8, 50 mM glycine, 500 mM NaCl, 5% glycerol, 500 mM imidazole). Eluted protein was subjected to further purification on a Superdex Hiload 26/60 S75 gel filtration column (GE Healthcare) at a flow rate of 3.2 mL/min using Buffer C (20 mM Hepes pH 7.5, 150 mM NaCl) and collected into 8 mL fractions. After analysis by SDS-PAGE, those fractions containing no traces of other contaminating proteins were pooled and concentrated in a 10 KDa cutoff Millipore filter (Merck Millipore) and concentration was measured using a BCA assay (Thermo Fisher Scientific Inc., USA).

C. Enzyme assays

Purified THAS and purified SGD were used in all assays. Strictosidine was purified by preparative reverse phase HPLC and quantified using ^{1}H NMR. Strictosidine aglycone was generated *in situ* prior to addition of THAS by incubation of strictosidine

and SGD in the appropriate solution for ten minutes, at which time strictosidine was completely converted to the aglycone. Steady state kinetic analyses were performed with 50 nM of THAS and 6 nM of SGD, 50 mM phosphate buffer (pH 7.5), 200 µM NADPH and an internal standard (50 µM caffeine). SGD has been shown to have a pH optimum between 6 and 8.5 (Luijendijk et al., 1998) and therefore should exhibit optimal activity at pH = 7.5. The kinetics were performed as follows: varying concentrations of strictosidine was placed in the wells of a 96-well plate with 50 mM phosphate buffer, followed by addition of 6 nM of SGD and the necessary volume of MilliQ water to standardize the volume. Caffeine (50 µM) was added to this mix as an internal standard. Another set of wells was prepared that contained pre-mixed solutions of 50 nM of THAS and 200 µM of NADPH. After 10 minutes of incubation with SGD, the strictosidine mix was added to the THAS + NADPH mix and mixed by pipetting several times. At 0.5 minutes, one minute and two minutes, a 10 µL aliquot of the reaction was placed in 80 µL H_20 + 0.1% formic acid premixed with 10 µL of methanol for a 10-fold final dilution of the sample. The 96-well plate was centrifuged at 4000 rpm for 10 min to pellet the enzyme precipitate and then analyzed by LCMS. A similar procedure was used to determine the K_m for NADPH, using 300 µM strictosidine and varying the NADPH concentration. The initial rate of the reaction (V_0) was calculated by fitting a linear regression through the points of product formation plotted against time. Michaelis-Menten plots were performed using SigmaPlot (Systat Software Inc.).

D. UPLC-QqQ-MS/MS analysis of the enzyme assays

Ultraperformance liquid chromatography (UPLC) was performed on a Waters Acquity UPLC system (Milford, MA) consisting of a binary pump, an online vacuum degasser, an autosampler, and a column compartment.

Kinetic studies and reaction monitoring

Chromatography was performed a BEH Shield RP18 (50 x 2.1 mm; 1.7 µm) column (Waters). The solvents used were H_2O + 0.1% formic acid as Solvent A and 100% acetonitrile as Solvent B, with a flow rate of 0.6 mL/min. Injection volume was 2 μL. The gradient profile was 0 min, 5% B; from 0 to 3.5 min, linear gradient to 35% B; from 3.5 min to 3.75 min, linear gradient to 100% B; wash at 100% B for 1 min; from 4.75 min to 6 min, back to 5% B for 1 min to re-equilibrate the column.

Quantification of tetrahydroalstonine: ajmalicine ratio

Separation of tetrahydroalstonine from ajmalicine could be achieved on a Luna NH2 (100 x 2.0 mm; 3 µm) column (Phenomenex). The chromatography was performed in HILIC mode using isocratic elution with 1% Solvent A and 99% Solvent C (50% acetonitrile + 50% isopropanol +0.1% formic acid) at a flow rate of 0.350 mL/min. The injection volume of both the standard solutions and the samples was 1 µL.

Mass spectrometry detection was performed on a Waters Xevo TQ-S mass spectrometer (Milford, MA, USA) equipped with an electrospray (ESI) source. Capillary voltage was 2.5 kV in positive mode; the source was kept at 150 °C; desolvation temperature was 500 °C; cone gas flow, 50 L/h; and desolvation gas flow, 900 L/h. Unit resolution was applied to each quadrupole.

Targeted methods for each compound were developed using either commercial standards (caffeine, ajmalicine and tetrahydroalstonine were purchased from Sigma-Aldrich) or enzymatically produced compounds (strictosidine and strictosidine

aglycone). Flow injections of each individual compound were used to optimize the MRM conditions. This was done automatically using the Waters Intellistart software. A minimum dwell time of 25 ms was applied to each MRM transition.

Four transitions were used to monitor the elution of tetrahydroalstonine and ajmalicine: *m/z* 353.2 > 117.0 (Cone 50, Collision 46), *m/z* 353.2 > 144.0 (Cone 50, Collision 26), *m/z* 353.2 > 210.1 (Cone 50, Collision 20) and *m/z* 353.2 > 222.0 (Cone 50, Collision 20). Transition *m/z* 353.2 > 144.0 was used for quantification of these two compounds. Transitions *m/z* 195.2 > 110.1 (cone 36, Collision 22) and *m/z* 195.2 > 138.2 (cone 36, Collision 18) were used for caffeine; transitions *m/z* 351.3 > 144.1 (cone 28, Collision 24) and *m/z* 351.3 > 170.2 (cone 28, Collision 22) were used for deglycosylated strictosidine; transitions *m/z* 531.3 > 144.1 (cone 32, Collision 36) and *m/z* 351.3 > 352.2 (cone 32, Collision 24) were used for detection of strictosidine.

For quantification of tetrahydroalstonine and ajmalicine, calibration curves were prepared and analyzed using nine calibrators in a range 2-500 ng/mL, in which the response was linear (R^2= 0.999 for both compounds). *In vitro* reactions were quenched by addition of 1 volume of methanol and dried under vacuum. The samples were then re-dissolved in Solvent C, appropriately diluted and analyzed by UPLC/QqQ-MS/MS. Data processing was done using MassLynx 4.1 and TargetLynx software (Waters).

<u>E. Large-scale product purification</u>

A large-scale reaction was setup for production of the THAS product: 4.3 mg of strictosidine was diluted in 20 mL of distilled water (400 µM), 500 µM NADPH, 3 nM of SGD, 100 nM of THAS, 20 units of Glucose-6-Phosphate Dehydrogenase (Roche

Diagnostics), and 1 mM of glucose-6-phosphate was added to the reaction mix and the pH was adjusted to 7.5 using 50 mM phosphate buffer. The reaction was incubated with gentle shaking at room temperature and reaction progress was assessed by subjecting aliquots to LCMS analysis. After 5 hours the reaction reached completion, and was quenched by addition of two volumes of methanol. The reaction was concentrated by evaporating to dryness under vacuum. The dried precipitate was extracted with methanol (1 mL) and the supernatant transferred to a clean round-bottom flask. Extraction of the precipitate was repeated until the extraction volume was approximately 15 mL. To the remaining precipitate was added 2 mL of water, followed by 2 mL of ethyl acetate (HPLC grade). The fractions were mixed gently and allowed to separate. Extraction of the water fraction with ethyl acetate was repeated 5 times, and the combined organic fractions were dried under vacuum and then resuspended in 50 µL of ethyl acetate. This was loaded onto a preparative silica TLC plate (UNIPLATE, Analtech™) that was pre-soaked with triethylamine (TEA). The TLC plate was developed in ethyl acetate : hexanes : TEA (24 : 75 : 1), twice, and allowed to air dry between runs. Visualisation of the bands was performed using UV (254 nm). A minor product with *m/z* 353, which had an identical R_f value to ajmalicine, was also observed, but could not be isolated in sufficient quantities for NMR.

F. NMR characterization

The major product of the reaction was excised from the TLC plate with a scalpel, and the silica was extracted with 2 mL of ethyl acetate five times. The ethyl acetate was filtered through filter paper to remove the silica. After drying under vacuum, the

product was resuspended in 200 µL of fresh ethyl acetate and passed through an SP column that was pre-equilibrated with hexanes (HPLC grade). The product was eluted using increasing amounts of ethyl acetate in hexanes and the first 3 elution fractions (25-75% ethyl acetate) were pooled and dried under vacuum. The sample was prepared for ^{1}H NMR by drying on a high-vacuum pump and resuspending in 300 µL of $CDCl_3$ (Sigma) and transferring to a Shigemi tube. NMR spectra (^{1}H NMR, ^{13}C NMR) were acquired using a Bruker Avance III 400 NMR spectrometer, operating at 400 MHz for ^{1}H and 100 MHz for ^{13}C. The residual ^{1}H- and ^{13}C NMR signals of CD_3Cl (δ 7.26 for ^{1}H and δ 77.36 for ^{13}C) were used as internal chemical shift references. The spectra corresponded to an authentic standard of tetrahydroalstonine (Sigma).

G. Virus Induced Gene Silencing

The THAS silencing fragment was amplified with primers 5’-GAGGTAACTGAGGTTGGCAGC-3’ and 5’-CATAGCCGGTCTAGGAGGA-3’, to give a gene fragment of 330 bp. The resulting fragment, when subjected to a tnBLAST search against the *C. roseus* transcriptome at http://medicinalplantgenomics.msu.edu, did not show greater than 75% sequence identity to any other gene, suggesting that cross-silencing is unlikely. This fragment was cloned into the pTRV2u vector and was used to silence the tetrahydroalstonine synthase in *C. roseus* seedlings. Leaves from the first two pairs to emerge following inoculation were harvested from eight plants transformed with the empty pTRV2u and pTRV2u-THAS. The collected leaves were frozen in liquid nitrogen, powdered using a

pre-chilled mortar and pestle, and subjected to LCMS and qRT-PCR analysis. Each pair of plant leaves was analyzed separately, for a total of 8 biological replicates.

H. LCMS analysis of silenced tissue

The alkaloid content of silenced leaves was determined by LCMS. Leaves were weighed and collected into a fixed volume of methanol (200 µL) and incubated at 56˚C for 60 min. After a 30-min centrifugation step at 5,000*g*, an aliquot of the supernatant (20 µL) was diluted to 400 µL with water and analyzed on a Shimadzu LCMS-IT-TOF Mass Spectrometer. The chromatographic separation was carried out on a Phenomenex Kinetex column 2.6u XB-C18 100 Å (100 × 2.10 mm, 2.6 µm), and the binary solvent system consisted of Solvent A, H_2O + 0.1% formic acid, and Solvent B, acetonitrile, at a constant flow rate 600 µL/min. The LC gradient began with 10% Solvent B and linearly increased to 30% Solvent B in 5 min, then increased to 90% B in 1 min, held for 1.5 min and brought back to 10% Solvent B. Peak areas were calculated using the Shimadzu Profiling Solution software and normalized by leaf mass (fresh weight).

The diastereomers tetrahydroalstonine and ajmalicine, which are both naturally present in *C. roseus* seedlings, do not separate under these LCMS (reverse phase) conditions. While ajmalicine and tetrahydroalstonine can be separated under HILIC chromatography conditions (see Section D), resolution of the complex mixture in seedling extracts has not yet been achieved. However, VIGS tissue did not show a decrease in the ajmalicine-derived compound serpentine, suggesting, albeit indirectly, that ajmalicine production is not substantially affected by this VIGS experiment (Fig. S2D).

I. Quantitative real-time PCR

RNA extraction was performed using the RNeasy Plant Mini Kit (Qiagen). RNA (1 µg) was used to synthesize cDNA in 20 µL reactions using the iScript cDNA Synthesis Kit (Bio-Rad). The cDNA served as template for quantitative PCR performed using the CFX96 Real Time PCR Detection System (Bio-Rad) using the SSO Advanced SYBR Green Supermix (Bio-Rad). Each reaction was performed in a total reaction volume of 20 µL containing an equal amount of cDNA, 0.25 mM forward and reverse primers, and 1x SsoAdvanced SYBRGreen Supermix (Bio-Rad). The reaction was initiated by a denaturation step at 95˚C for 10 min followed by 41 cycles at 95˚C for 15 s and 60˚C for 1 min. Melting curves were used to determine the specificity of the amplifications. Relative quantification of gene expression was calculated according to the delta-delta cycle threshold method using the 40S ribosomal protein S9 (RPS9). The primers 5'-TTGAGCCGTATCAGAAATGC-3' and 5'-CCCTCATCAAGCAGACCATA-3' were used for RPS9, and 5'-TGACAGTGATTAGTTCTTCACTTAAGA-3' and 5'-TGCACCCTGCATTTCTTC-3' were used for THAS. All primer pair efficiencies were between 98% and 108%, and the individual efficiency values were considered in the calculation of normalized relative expression, which was performed using the Gene Study feature of CFX Manager Software. All biological samples were measured in technical duplicates.

J. Isothermal Titration Calorimetry (ITC)

Titration was performed using a MicroCal iTC200 System (GE Healthcare Life Sciences) by injecting 2 µL of titrant at intervals of 110 s. The first injection was 0.5

µL and not used for data analysis. Titrations were carried out at 30˚C, and stirring speed was 700 rpm. For determination of the dissociation constant of THAS and NADPH, THAS was dialyzed overnight against 2 L of Buffer C (20 mM Hepes (pH 6.8), 100 mM NaCl). The protein concentration was adjusted with dialysis buffer before the experiment. THAS dimer (40 *µ*M) was titrated with 1 mM of NADPH (dissolved in Buffer C). Titrations were carried out in triplicate and a control titration was carried out in which Buffer C was injected into the cell containing THAS in order to determine the dilution or mixing heat. This heat was then subtracted from the analysis of the NADPH titrations. Data analysis was done using Origin 7.0 software (MicroCal) by fitting a single site model to the data obtained. The dissociation constant, K_d, binding enthalpy (ΔH), and entropy (ΔS) was determined for each replicate. Titration with strictosidine aglycone (also in Buffer C) did not give significant binding heat, which indicates it cannot bind to the active site before binding of NADPH.

K. Pull down of THAS with SGD

Purified THAS was prepared for pull-down assay by cleaving the His-tag using 3C protease. THAS (200 µg) was incubated with 3C protease (1 µg) overnight at 4˚C, then 1 µg of fresh 3C protease was added and the reaction was allowed to progress for another thirty minutes at room temperature.

To purify the cleaved THAS, the reaction was passed through a 0.5 mL Ni-NTi Agarose slurry (Qiagen Ltd., Manchester, UK) pre-equilibrated with Buffer D (20 mM Hepes (pH 7.5), 150 mM NaCl). The flow through was collected and an aliquot was analyzed by SDS-page gel to verify the molecular weight. Cleaved THAS was

concentrated using a Millipore filter unit with a 10 KDa cutoff and the concentration measured using a BCA assay. A glass chromatography column (0.5 cm x 10 cm) was loaded with 0.5 mL of Ni-NTi Agarose slurry (Qiagen) that was washed and equilibrated with 15 mL of Buffer D. His-tagged SGD (380 µg) was loaded onto the column and 1 mL fractions were collected. The column was then washed with 5 mL of Buffer C, followed by loading of 100 µg of THAS, premixed with 0.5 mM of NADPH. The column was washed with 5 mL of Buffer D and elution was carried out with 3 mL of buffer E (20 mM Hepes (pH 7.5), 150 mM NaCl, 250 mM imidazole). Aliquots (20 *µ*L) of each fraction were analyzed by SDS-page gel and stained using InstantBlue (Expedeon Ltd, Cambridgeshire, UK).

L. Subcellular localization, NLS mutation and analysis of protein-protein interactions using bimolecular fluorescent complementation (BiFC) assays

The full-length ORF of THAS was amplified using the specific primers 5'-CTGAGAACTAGTATGGCAATGGCTTCAAAGTCAC-3' and 5'-CTGAGAACTAGTATTTGATTTCAGAGTGTTCCCTATATCAATC-3', which were designed to introduce the *Spe*I restriction site at both cDNA extremities. The PCR product was sequenced and cloned at the 5' end of the yellow fluorescent protein (YFP) coding sequence to generate the THAS-YFP fusion or at the 3' end to express the YFP-THAS fusion. Mutation of the nuclear localization sequence (NLS) of THAS (KKKR, residues 214-217) into residues NTSG was achieved by amplification of the coding sequence of the first 213 residues with primers 5'-CTGAGAAGATCTATGGCAATGGCTTCAAAGTCAC-3' and 5'-CTGAGAACTAGTATTAAGTGAAGAACTAATCACTGTCACCTT-3', introducing *Bgl*II

and *Spe*I restriction sites at the 5' and 3' extremities of the resulting cDNA. This PCR product was cloned via *Bgl*II and *Spe*I into the pSCA-cassette YFPi plasmid, in order to create the pSCA-THAS-213 vector. The remaining part of the THAS sequence (encoding residues 218-356) was amplified with primers 5'-CTGAGAACTAGTGGTGAAGCCTTTGAGAAATTCGGA-3' and 5'-CTGAGAACTAGTATTTGATTTCAGAGTGTTCCCTATATCAATC-3', introducing *Spe*I restriction at both cDNA extremities, which were used to clone the resulting PCR product into the pSCA-THAS-213 vector to express the ΔNLS-THAS-YFP fusion.

Transient transformation of *C. roseus* cells by particle bombardment and fluorescence imaging were performed following the procedures previously described (Guirimand et al., 2009; Guirimand et al., 2010). Briefly, *C. roseus* plated cells were bombarded with DNA-coated gold particles (1 μm) and 1,100 psi rupture disc at a stopping-screen-to-target distance of 6 cm, using the Bio-Rad PDS1000/He system. Cells were cultivated for 16 h to 38 h before being harvested and observed. The subcellular localization was determined using an Olympus BX-51 epifluorescence microscope equipped with an Olympus DP-71 digital camera and a combination of YFP and CFP filters. The pattern of localization presented in this work is representative of *circa* 50 observed cells. The nuclear or nucleocytosolic localizations of the different fusion proteins were confirmed by co-transformation experiments using the nuclear-CFP marker and the nucleocytosolic CFP marker (Guirimand et al., 2010). Such plasmid co-transformations were performed using 400 ng of each plasmid or 100 ng for BiFC assays. Plasmids encoding bZIP63-YFP^{N} and bZIP63-YFP^{C} were used as controls (Waadt et al., 2008).

The plasmid expressing the LAMT-YFP^{C} fusion protein, used to check the specificity of protein interactions in BiFC assays, has been described previously (Guirimand et al., 2011).

M. Supplemental References

Berrow, N.S., Alderton, D., Sainsbury, S., Nettleship, J., Assenberg, R., Rahman, N., Stuart, D.I., and Owens, R.J. (2007). A versatile ligation-independent cloning method suitable for high-throughput expression screening applications. Nucl Acid Res *35*, e45.

Guirimand, G., Burlat, V., Oudin, A., Lanoue, A., St-Pierre, B., and Courdavault, V. (2009). Optimization of the transient transformation of *Catharanthus roseus* cells by particle bombardment and its application to the subcellular localization of hydroxymethylbutenyl 4-diphosphate synthase and geraniol 10-hydroxylase. Plant Cell Rep *28*, 1215-1234.

Guirimand, G., Courdavault, V., Lanoue, A., Mahroug, S., Guihur, A., Blanc, N., Giglioli-Guivarc'h, N., St-Pierre, B., and Burlat, V. (2010). Strictosidine activation in Apocynaceae: towards a "nuclear time bomb"? BMC Plant Biol *10*, 182.

Guirimand, G., Guihur, A., Ginis, O., Poutrain, P., Héricourt, F., Oudin, A., Lanoue, A., St-Pierre, B., Burlat, V., Courdavault, V. (2011) The subcellular organization of strictosidine biosynthesis in Catharanthus roseus epidermis highlights several trans-tonoplast translocations of intermediate metabolites. FEBS J. *278*, 749-763.

Luijendijk, T.J.C., Stevens, L.H., and Verpoorte, R. (1998). Purification and characterisation of strictosidine β-D-glucosidase from *Catharanthus roseus* cell suspension cultures. Plant Physiol Biochem *36*, 419-425.

Waadt, R., Schmidt, L.K., Lohse, M., Hashimoto, K., Bock, R., and Kudla, J. (2008). Multicolor bimolecular fluorescence complementation reveals simultaneous formation of alternative CBL/CIPK complexes *in planta*. Plant J *56*, 505-516.
